# Adipsin alleviates cardiac microvascular injury in diabetic cardiomyopathy through Csk-dependent signaling mechanism

**DOI:** 10.1186/s12916-023-02887-7

**Published:** 2023-05-26

**Authors:** Xuebin Zhang, Yu Duan, Xiao Zhang, Mengyuan Jiang, Wanrong Man, Yan Zhang, Dexi Wu, Jiye Zhang, Xinglong Song, Congye Li, Jie Lin, Dongdong Sun

**Affiliations:** Department of Cardiology, Xijing Hospital, The Fourth Military Medical University, Xi’an, China

**Keywords:** Diabetic cardiomyopathy, Adipsin, Exosomes, Adherens junctions, Permeability

## Abstract

**Background:**

Microvascular complications are associated with an overtly increased risk of adverse outcomes in patients with diabetes including coronary microvascular injury which manifested as disruption of adherens junctions between cardiac microvascular endothelial cells (CMECs). However, particular mechanism leading to diabetic coronary microvascular hyperpermeability remains elusive.

**Methods:**

Experimental diabetes was induced in mice with adipose tissue-specific Adipsin overexpression (Adipsin^LSL/LSL^-Cre) and their respective control (Adipsin^LSL/LSL^). In addition, cultured CMECs were subjected to high glucose/palmitic acid (HG + PA) treatment to simulate diabetes for a mechanistic approach.

**Results:**

The results showed that Adipsin overexpression significantly reduced cardiac microvascular permeability, preserved coronary microvascular integrity, and increased coronary microvascular density. Adipsin overexpression also attenuated cardiac dysfunction in diabetic mice. E/A ratio, an indicator of cardiac diastolic function, was improved by Adipsin. Adipsin overexpression retarded left ventricular adverse remodeling, enhanced LVEF, and improved cardiac systolic function. Adipsin-enriched exosomes were taken up by CMECs, inhibited CMECs apoptosis, and increased CMECs proliferation under HG + PA treatment. Adipsin-enriched exosomes also accelerated wound healing, rescued cell migration defects, and promoted tube formation in response to HG + PA challenge. Furthermore, Adipsin-enriched exosomes maintained adherens junctions at endothelial cell borders and reversed endothelial hyperpermeability disrupted by HG + PA insult. Mechanistically, Adipsin blocked HG + PA-induced Src phosphorylation (Tyr416), VE-cadherin phosphorylation (Tyr685 and Tyr731), and VE-cadherin internalization, thus maintaining CMECs adherens junctions integrity. LC-MS/MS analysis and co-immunoprecipitation analysis (Co-IP) unveiled Csk as a direct downstream regulator of Adipsin. Csk knockdown increased Src phosphorylation (Tyr416) and VE-cadherin phosphorylation (Tyr685 and Tyr731), while abolishing Adipsin-induced inhibition of VE-cadherin internalization. Furthermore, Csk knockdown counteracted Adipsin-induced protective effects on endothelial hyperpermeability in vitro and endothelial barrier integrity of coronary microvessels in vivo.

**Conclusions:**

Together, these findings favor the vital role of Adipsin in the regulation of CMECs adherens junctions integrity, revealing its promises as a treatment target against diabetic coronary microvascular dysfunction.

**Graphical Abstract:**

Graphical abstract depicting the mechanisms of action behind Adipsin-induced regulation of diabetic coronary microvascular dysfunction.

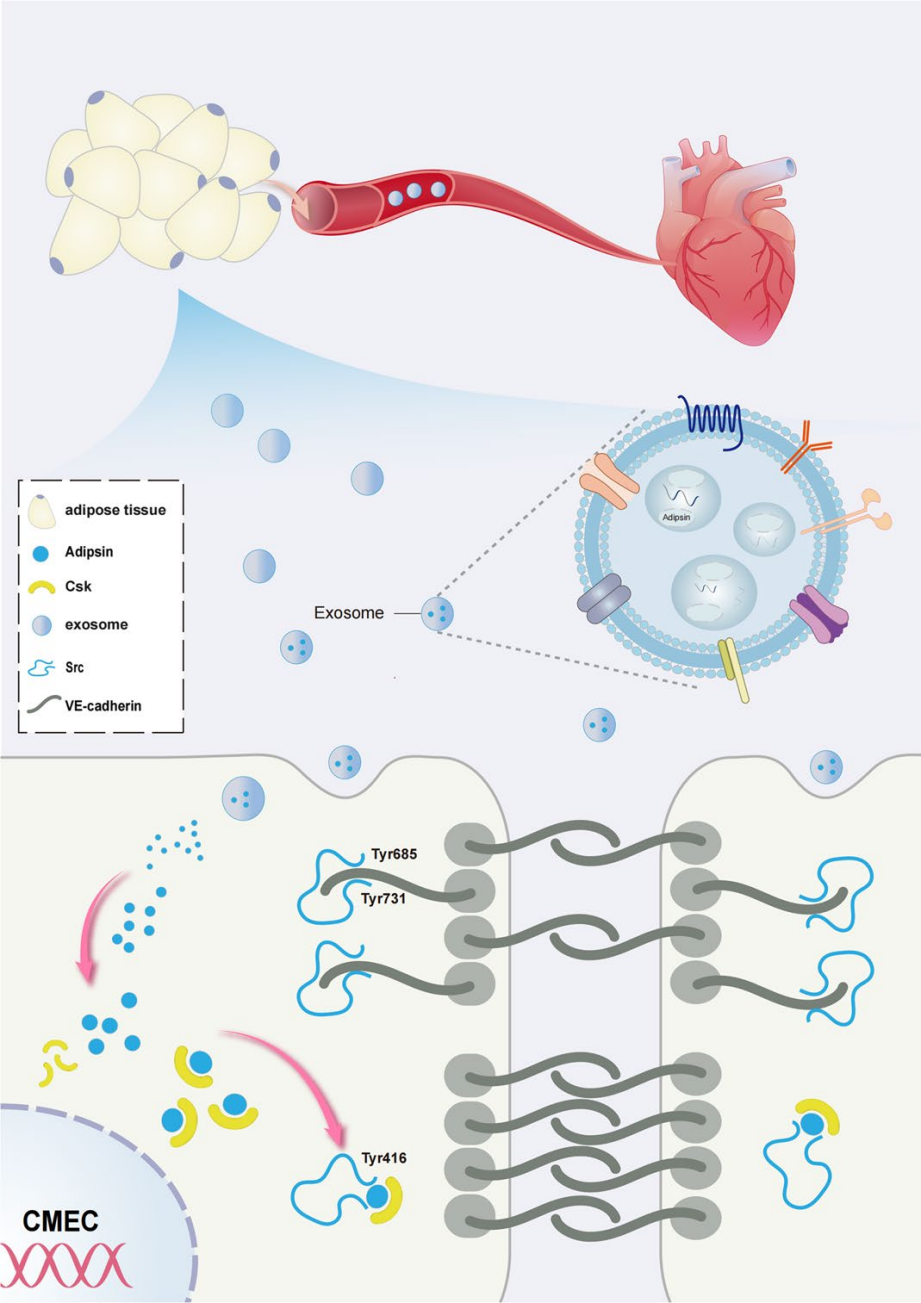

**Supplementary Information:**

The online version contains supplementary material available at 10.1186/s12916-023-02887-7.

## Highlights

1. Adipsin is significantly downregulated in diabetes.

2. Adipose tissue-specific Adipsin overexpression alleviates coronary microvascular injury, and enhances cardiac diastolic and systolic function.

3. Adipsin-enriched exosomes inhibit VE-cadherin phosphorylation and reverse VE-cadherin internalization, thus maintaining the integrity of CMEC adherens junctions.

4. Csk is a downstream regulator of Adipsin-evoked protection against microvascular hyperpermeability by inhibiting Src activity.

**Supplementary Information:**

The online version contains supplementary material available at 10.1186/s12916-023-02887-7.

## Background

Diabetes mellitus is a non-communicable chronic disease worldwide, which is estimated that 8.8% of adult populations were afflicted in 2015. In 2040, this population is estimated to substantially hike to 10.4%, translating to 642 million diabetic patients [1]. According to the epidemiological data of the World Health Organization, diabetes mellitus and its complications are responsible for 1.5 million mortalities worldwide each year, imposing an enormous public health threat [2]. In recent years, microvascular complications of diabetes are gaining much attention since these pathological changes elevate a composite endpoint of cardiovascular death or heart failure hospitalization by 18% [3]. Microvascular complications are highly prevalent in patients with diabetes, including chronic kidney disease, retinopathy, and peripheral neuropathy [4–6]. Noteworthy, functional and structural microvascular alterations represent early events of more widespread derangement of coronary circulation in diabetic patients [7–9]. Early recognition of diabetic microvascular complications is essential, as up to 25% of newly diagnosed diabetic patients may have already developed one or more microvascular complications [10]. However, pathogenesis of cardiac microvascular complications is not well understood in diabetes. Interventions capable of reversing the pathophysiologic cues in the progression of cardiac microvascular complications of diabetes are needed.

Adipokines are secreted proteins mainly produced by adipocytes and released to the circulatory system to communicate with different organs with regard to the functional status of adipose tissues. In consequence, adipokines are involved in a plethora of physiological functions, including insulin sensitivity and secretion, glucose and lipid metabolism, endothelial function, and cardiomyocyte survival [11]. This wide range of pathophysiological properties suggests a possible role of adipokines as pathological biomarkers for various anomalies including diabetes mellitus. As the first described adipokine, Adipsin plays a cardinal role in metabolic homeostatic regulation in diabetes [12]. Adipsin is a member of the serine protease gene family, secreted mainly by adipose cells into peripheral circulation [13, 14]. Paradoxically, Adipsin mRNA levels were profoundly decreased in *db/db* and *ob/ob* mice [15]. Lo and colleagues reported that chronic replenishment of Adipsin ameliorated hyperglycemia, increased insulin levels while preserving beta cells by blocking dedifferentiation and death in diabetic *db/db* mice. Higher levels of circulating Adipsin were associated with a significantly low risk of developing diabetes in adults [16].

However, the precise function of Adipsin in relation to diabetic microvascular complications remains elusive. To this end, this study was designed to investigate the potential role of Adipsin in the regulation of coronary microvascular complications, as well as the underlying mechanism involved.

## Methods

### Human studies

The studies involving participants were reviewed and approved by the Institutional Review Board of Xijing Hospital. Written informed consent was obtained from all participants. Serum samples were collected from healthy individuals and type 2 diabetic patients. According to the manufacturer's instructions, serum Adipsin levels were quantified using an ELISA kit (Elabscience Biotechnology Co., Ltd, E-EL-H6007).

### Animal studies

All experimental animal procedures were approved by the Animal Care and Use Committee of the Fourth Military Medical University and followed the Animal Research Advisory Committee of the National Institutes of Health guidelines. Adipsin^LSL/+^ mice and Adipoq-Cre (Cre) mice were purchased from Shanghai Southern Model Biotechnology Co., Ltd. Adipose tissue-specific Adipsin overexpression mice (Adipsin^LSL/LSL^-Cre) were constructed using Cre recombinase-mediated excision of a STOP cassette (Additional file 1: Fig. S1). Genotypes were confirmed by genomic PCR according to the manufacturer's procedure (Bimake, B40015). Male mice were used for all experiments.

High-fat diet (Research Diets, D12492) and low-dose injections of streptozotocin (STZ, 50 mg/kg, Sigma-Aldrich, S0130) dissolved in 0.1 mol/l citrate buffer (pH 4.2–4.5) for 5 consecutive days were used to induce an animal model of type 2 diabetes mellitus (DM) as described with further optimization [17–19]. Furthermore, the non-diabetic group (Non-DM) received a standard chow diet and an intraperitoneal injection with an equal volume of citrate buffer. One week after the final STZ injection, mice that failed to meet the criteria (fasting blood glucose levels, 16.6 mmol/L) of diabetes diagnosis were given an additional injection of STZ. Four groups were assigned as follows: Non-DM + Adipsin^LSL/LSL^, Non-DM + Adipsin^LSL/LSL^-Cre, DM + Adipsin^LSL/LSL^, and DM + Adipsin^LSL/LSL^-Cre. The glucose tolerance test was performed using intraperitoneal injection of glucose following 16h-fasting, and the insulin resistance tolerance test was performed using intraperitoneal injection after 2-h fasting in mice. Blood glucose levels were measured using blood samples from the tail vein at the indicated time points.

Csk knockdown was achieved by intramyocardial injection of Adeno-associated virus 9 (Tsingke Biotechnology Co., Ltd) vector carrying Csk shRNA in vivo as described previously [20, 21]. Detailed information was provided in the Additional file 1: Fig. S2. In brief, mice were anesthetized with 2% isoflurane inhalation and secured on the shelf. Anesthetized mice were mechanically ventilated before aseptic left thoracotomy. The AAV9-Csk-shRNA or scramble (2.0+E10.0 viral genome particles per mouse) was injected into the left ventricular wall of transgenic mice at 3 different sites using the Hamilton micro-syringes. Exosomes were delivered via tail vein injections as follows. A total of 200 µL exosomes (1.0+E10.0 particles/kg) was administered once a week after 8 weeks of high-fat diet feeding until the termination of the experiment [22]. Equal volumes of PBS were injected as the vehicle control. At the end of the experiment, mice were euthanized using 30% VD/minof 100% carbon dioxide in adherence to the 2020 AVMA Guidelines on Euthanasia.

### Echocardiography

Mice were anesthetized with 2% isoflurane inhalation and were placed in supine position on a heated plate at 37 °C. The chest and upper abdomen were debrided to expose the skin thoroughly, and cardiac function was evaluated by transthoracic echocardiography (VisualSonics, Vevo2100). The left ventricular diastolic function was assessed by the measurement of early peak flow velocity (E) to late peak flow velocity (A) wave ratio. The left ventricular systolic function was indicated by measurement of the left ventricular internal diameter at end-diastole (LVIDd) and left ventricular internal diameter at end-systole (LVIDs). Each measurement was obtained by calculating the average result of five consecutive heartbeats.

### Enzyme-linked immunosorbent assay (ELISA)

Serum Adipsin levels were detected using the Mouse Adipsin ELISA Kit according to the manufacturer's procedure (Elabscience Biotechnology Co., Ltd, E-EL-M0335c). The optical density (OD value) was determined at the wavelength of 450 nm using a spectrophotometer.

### Isolation of mouse primary cardiac microvascular endothelial cells

CMECs were isolated as described with appropriate optimization methods [23, 24]. Briefly, left ventricles were isolated from mice under general anesthesia (2% isoflurane inhalation). The heart was washed with ice-cold PBS and cut into 1 mm^3^ size, digested with 0.2% collagenase II at 37°C for 10 min until tissue pieces became gelatinous. Then digestion was continued with 0.25% trypsin at 37°C for 10 min, filtered through a 70-μm filter, and centrifuged at 1000g for 5 min. After removal of the supernatant, the pellet was resuspended in culture flasks with a complete medium containing 20% fetal bovine serum. CMECs were differentiated using the DiI-Ac-LDL marker, and cell proportions more significant than 90% were used for subsequent experiments. The diabetic group (HG + PA) was exposed to 25 mM glucose and palmitic acid (PA, 300 μM) to mimic diabetes. The control group (NG + vehicle) was given 5 mM glucose and solvent control. Mannitol at the same concentration was used as osmolarity control.

### Isolation and identification of adipose tissue-derived exosomes

Subcutaneous and epididymal adipose tissues were obtained from Adipsin^LSL/LSL^ and Adipsin^LSL/LSL^-Cre mice under general anesthesia (2% isoflurane inhalation). The tissue blocks were cut to approximately 1×1×1 mm size following rinsing in PBS on a sterile operating table, and the supernatant was collected after 24h of incubation in the exosomes-free medium. Exosomes were isolated from supernatant according to the conventional exosome isolation protocol [25]. Briefly, cells and impurity debris were removed from supernatants by centrifugation at 500g for 10min, 2000g for 10min, and 10,000g for 30min. The supernatant was filtered with a sterile 0.22-μm filter, and exosomes were isolated by ultracentrifugation at 100,000g for 70min. Exosomes in plasma were isolated using the commercial kit (Invitrogen, 4484450) according to the manufacturer’s protocol. The harvested exosomes were identified by transmission electron microscopy (JEM-2000EX) and Nanoparticle tracking analysis (Nanosight-NS300).

### Scanning electron microscopy

Cardiac microvascular casting was employed to assess the integrity of microvascular endothelial cells as previously described [23, 24]. Low-viscosity resin mixed with benzoyl peroxide was perfused through the aorta and was then immersed in a 5% sodium hydroxide solution at room temperature. Connective tissues around the vessels were removed after 3–4 days. Isolated cardiac microvessels were dehydrated, dried, plated, and observed with a scanning electron microscope (HITACHI, S4800).

### Transmission electron microscopy

The cardiac microvascular structure was visualized using standard transmission electron microscopy. Myocardial tissues were fixed with 2.5% glutaraldehyde overnight, washed, dehydrated, penetrated, and polymerized following lanthanum nitrate perfusion. The resin-embedded samples were imaged and captured using transmission electron microscopy (HITACHI, FE-2000).

### Permeability assay in vitro

As previously reported [26], endothelial permeability was evaluated using 6.5-mm Transwell® 0.4-µm pore size polycarbonate membrane chambers (Corning, 3413). Briefly, CMECs were inoculated and changed to 70 kDa FITC-labeled dextran (Sigma-Aldrich, 46945) after growing into the monolayer. Fluorescence intensity was measured using a plate reader with filters for 485 nm excitation and 535 nm emission.

### Trans-endothelial electrical resistance (TEER)

Trans-endothelial electrical resistance (TEER) analysis was performed to monitor the monolayer integrity as described previously [26]. In brief, CMECs were cultured as monolayers on semi-permeable filters (Corning, 3413) and exposed to NG + vehicle or HG + PA treatment. TEER was measured using EVOM Volt-Ohm Meter (World Precision Instruments Inc) at the indicated points. The percentage of TEER change was calculated for statistical analyses.

### siRNA transfection

CMECs were cultured to a 60-80% confluence in a 6-well plate. Then cells were transfected with a siRNA against Csk or a negative control siRNA (Tsingke Biotechnology Co., Ltd). According to the manufacturer’s instruction, siRNA diluted in Lipofectamine 2000 was premixed with Opti-MEM media (50nM) at room temperature for 15min and was incubated with cells for 24h or 48 h for further study.

### Co-immunoprecipitation (Co-IP)

Cell lysates were obtained following digestion with a RIPA buffer (Beyotime, P0013B). Then an immune complex was formed by binding the target protein with the specific antibody and co-incubation with the Pierce Protein A/G Magnetic Beads. Finally, the antibody/protein complex was separated from other cellular proteins by magnetic or centrifugal methods. Samples are electrophoresed on SDS-PAGE gels.

### Liquid chromatography-mass spectrometry analysis (LC-MS)

The experiment was performed with the support of Applied Protein Technology. Briefly described as follows: samples were lysate to extract proteins before quantification using the BCA method. Each sample was separated by NanoElute, an HPLC liquid phase system with a nanoliter flow rate. Buffer A was 0.1% formic acid aqueous solution, and B was 0.1% formic acid acetonitrile aqueous solution. The chromatographic column was equilibrated with 95% of liquid A. Samples were loaded onto the loading column by the autosampler and separated by the analytical column at a flow rate of 300 nL/min. Samples were separated and analyzed using a timsTOF Pro mass spectrometer. Protein identification and quantitative analysis were performed using mass spectrometry assisted by the MaxQuant software.

### Wound healing scratch assay

The migration ability of cells was assayed using the wound-healing scratch assay. In brief, CMECs were inoculated in a 6-well plate. When the cells were fully plated, a straight line was drawn at the bottom of the well. Then, the culture was continued with a serum-free or low-serum medium, and photographs of cell scratches were taken at selected appropriate time points. The migration distance of cells was determined by the ratio of the 12-hour scratch width to the 0-hour scratch width.

### Cell migration assay

Cell migration ability was evaluated using 6.5-mm Transwell 8.0-µm pore-sized polycarbonate membrane chambers (Corning, 3422). First, 200 µl of serum-free medium cell suspension was inoculated in the upper chamber, and 600 µl of medium containing 20% FBS was added in the lower chamber. CMECs were then incubated for 24 h, fixed with 4% paraformaldehyde, and stained with 0.1% crystal violet (Beyotime, C0121). Finally, non-migrated cells in the upper chamber were gently wiped with a cotton swab, and migrated cells were observed with an Olympus microscope.

### Tube formation assay

Endothelial cell tube formation experiments were performed in 250-µl Matrigel (Corning, 354248) coated 24-well plates. Inoculate 200 µl of cell suspension at a density of 2.0+E5 into a 24-well plate covered with Matrigel, and then continue to incubate for 12 h. After fixing with 4% paraformaldehyde at an appropriate time, the tube formation was visualized using microscopy.

### Flow cytometry

The Annexin V-FITC Apoptosis Assay Kit (Beyotime, C1062) was used to detect apoptosis according to the manufacturer’s manual. Briefly, CMECs were digested and centrifuged. Then 5-μl Annexin V-FITC and 10-μl propidium iodide staining solution were sequentially added. After incubation for 20 min at room temperature, apoptosis was detected using flow cytometry (Beckman Coulter, CytoFLEX S).

### TUNEL staining

TUNEL staining was performed according to the instructions of the Beyotime one-step TUNEL Apoptosis Detection Kit (Beyotime, C1088). Finally, nuclei were stained with DAPI (Beyotime, C1006), and images were collected by confocal fluorescence microscopy.

### CCK-8 proliferation assay

To assess cell viability, CMECs were plated and given indicated drug treatment in 96-well plates. Then cell viability was determined via the Cell Counting Kit-8 (Sigma, 96992) according to the manufacturer’s instructions. The absorbance at 450 nm was read using the microplate reader (Molecular Devices, SpectraMax M5).

### Immunofluorescence staining

CMECs or 10-μm frozen sections were fixed with 4% paraformaldehyde for 15min before incubation with an immunostaining permeabilization solution Triton X-100 (Beyotime, P0096) for 20min at room temperature and blocking with goat serum (Beyotime, C0265) for 1 h at room temperature. Then cells were incubated with primary antibody overnight at 4°C and incubated with fluorescent secondary antibody for 1 h at room temperature, and nuclei were stained with DAPI (Beyotime, C1006). Images were acquired by observing under a confocal fluorescence microscope. Antibody dilution information was shown in the Supplementary Materials (Additional file 1: Table S1).

### Quantification of blood vessel density

Anti-CD31 immunofluorescence intensity was used to determine blood vessel density in heart. The blood vessel density was analyzed by using the VesselJ plugin of ImageJ software as described [27].

### Quantitative real-time PCR

Total RNA was extracted using TRIzol™ reagent (Invitrogen, 15596018). RNA concentration was measured with the Nano Drop2000 spectrophotometer (Thermo Scientific). The cDNA was synthesized, and genomic DNA was removed using the PrimeScript™ RT reagent Kit with gDNA Eraser (Takara, RR047A). Quantitative analysis was performed using TB Green® Premix Ex Taq™ II (Takara, RR820A) and StepOnePlusTM Real-Time PCR (Thermo Fisher Scientific, 4376600). The primer sequence information was shown in the Supplementary Material (Additional file 1: Table S2).

### Western blot

Serum protein was extracted using the Serum Protein Extraction Kit (Bestbio, BB-31954). Tissue or cells were lysed with RIPA lysis buffer (Beyotime, P0013E) with protease inhibitor and phosphatase inhibitor. Protein concentration was measured using the BCA protein concentration assay kit (Beyotime, P0010). Then protein samples were separated in SDS-PAGE gels and transferred to PVDF membranes with 0.22-μm pore size. Then membranes were closed for 20 min by fast closure solution and incubated with primary antibody at 4°C overnight, followed by horseradish peroxidase (HRP)-labeled secondary antibody at room temperature for 1 h, using the Image Lab system for analysis. Antibody dilution information was shown in the Supplementary Material (Additional file 1: Table S1).

### Statistical analysis

Data analysis was performed using GraphPad Prism 9.0 software. Results were expressed as mean ± the standard error of the mean, and n indicates sample size of each group. Statistical analyses between groups were done by unpaired Student’s *t*‐test and one-way ANOVA followed by a Fisher’s post hoc comparison test. A *p*-value less than 0.05 was accepted to be a significant difference.

## Results

### Downregulation of Adipsin in mice and patients with type 2 diabetes

**Fig. 1:**
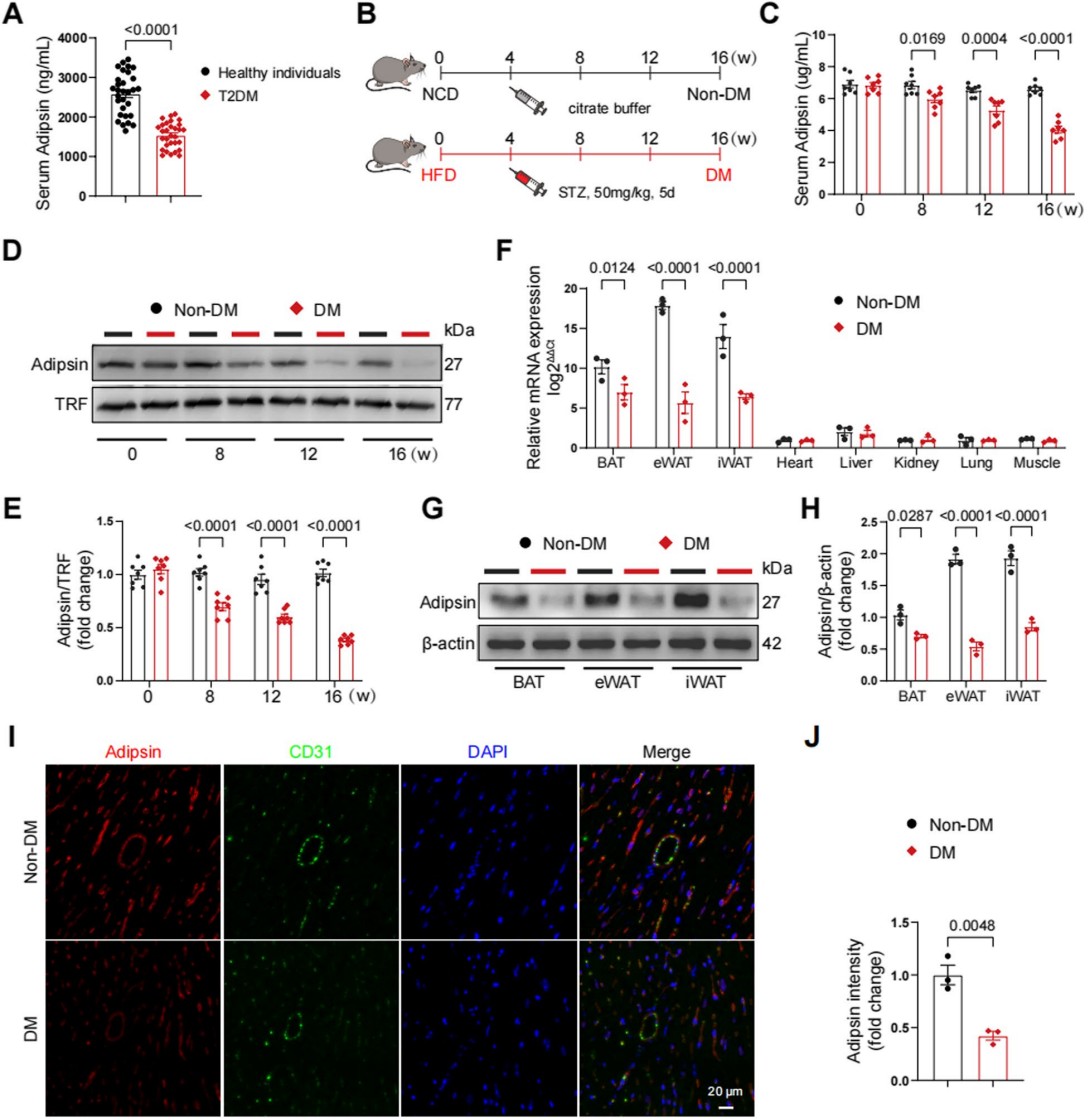
Adipsin is downregulated in diabetic mice. **A** Serum Adipsin levels detected using ELISA assay in healthy individuals and T2DM. **B** Schematic protocols of high-fat diet and streptozotocin-induced type 2 diabetic mouse model. Non-DM, non-diabetes; DM, diabetes mellitus. **C** Serum Adipsin levels determined using ELISA at indicated time points in mice. **D** Representative Western blot images of serum Adipsin levels at 0, 8, 12, and 16 weeks following high-fat diet feeding. **E** Quantitative analysis of serum Adipsin levels in **D**. **F** Adipsin mRNA levels quantified in various tissues. **G** Representative Western blot images of Adipsin in different types of adipose tissues. BAT, brown adipose tissue, eWAT; epididymal white adipose tissue; iWAT, inguinal white adipose tissue. **H** Quantitative analysis of Adipsin levels in **G**. **I** Representative immunofluorescence images of Adipsin around blood vessels in mice. Scale bar = 20 μm. **J** Quantitative analysis of Adipsin fluorescence intensity in **I**. Data were presented as mean ± SEM. Student’s *t*‐test was used for statistical analysis

To decipher the effect of Adipsin in a clinical context, serum samples were collected from type 2 diabetic patients and healthy individuals (Additional file 1: Table S3). Serum Adipsin levels were remarkably declined in diabetic patients (Fig. 1A). To gain insights into potential effect of Adipsin in diabetic mice, an animal model of type 2 diabetes was constructed utilizing a high-fat diet and low-dose STZ injection approaches (Fig. 1B). Serum samples were collected and analyzed by ELISA for Adipsin concentrations. Consequently, serum Adipsin levels were progressively decreased in a time-dependent manner in diabetic group (DM) compared with the non-diabetic group (Non-DM) (Fig. 1C). Concomitantly, similar findings were noted with assessment of serum Adipsin levels using Western blot analysis (Fig. 1D, E). Next, we monitored mRNA levels of Adipsin in brown adipose tissue (BAT), epididymal white adipose tissue (eWAT), inguinal white adipose tissue (iWAT), heart, liver, kidney, lung, and skeletal muscle (Fig. 1F). Our results indicated high Adipsin mRNA levels in BAT, eWAT, and iWAT. Diabetic challenge decreased Adipsin mRNA levels in these above tissues. Western blot analysis also revealed overtly decreased Adipsin expression in BAT, eWAT, and iWAT in diabetic conditions (Fig. 1G, H). Serum samples from mice were collected and analyzed by ELISA for Adipsin concentrations. Notably, immunofluorescence staining indicated overtly decreased Adipsin content in cardiac tissue under diabetic stress (Fig. 1I, J). These observations revealed that Adipsin was significantly downregulated in diabetes.

### Adipsin alleviates diabetes-induced myocardial microvascular injury

**Fig. 2:**
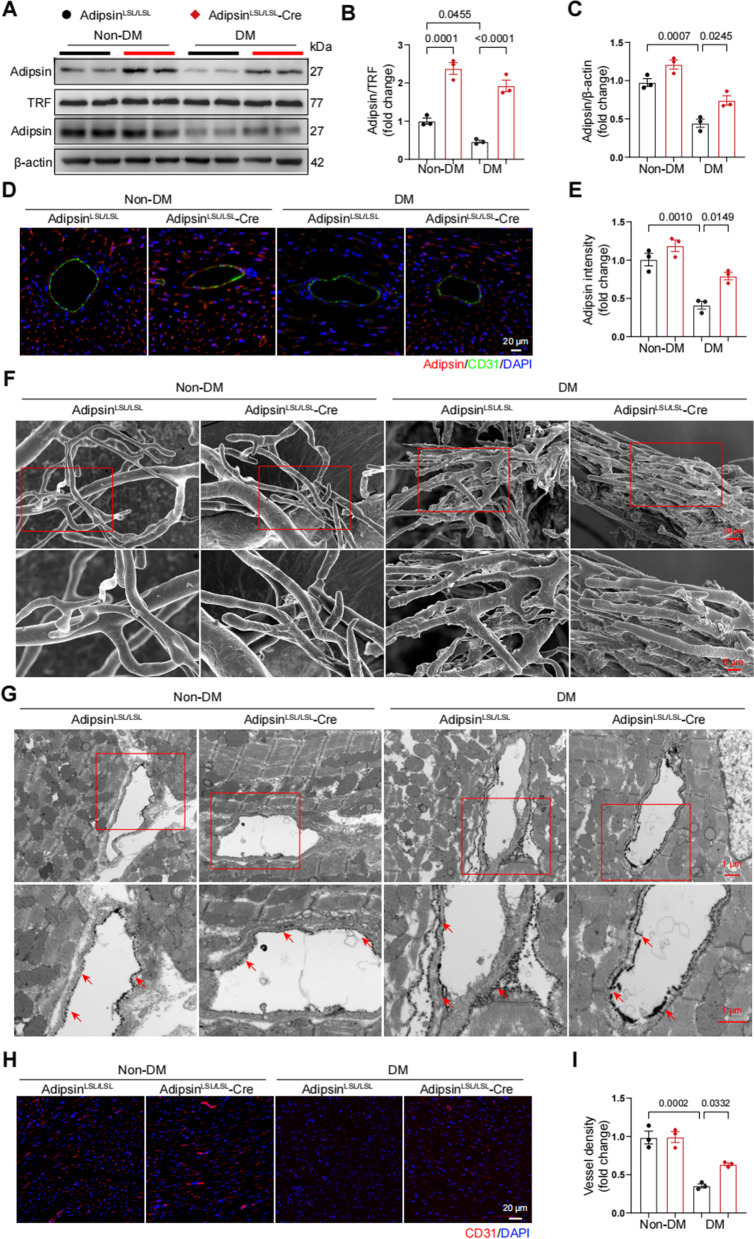
Adipose tissue-specific Adipsin overexpression improves microvascular dysfunction. **A** Representative Western blot images of Adipsin levels in serum and heart from different groups. **B** Quantitative analysis of serum Adipsin levels in **A**. **C** Quantitative analysis of Adipsin levels in cardiac tissues in **A**. **D** Representative immunofluorescence images of Adipsin around blood vessels. Scale bar = 20 μm. **E** Quantitative analysis of Adipsin fluorescence intensity in **D**. **F** Representative scanning electron micrographs of cardiac microvessels corrosion in different groups. Insert boxes indicated areas of magnification shown below. Scale bar = 10 μm (upper) and 5 μm (bottom). **G** Representative transmission electron micrographs of cardiac capillaries with lanthanum nitrate staining indicating barrier function. Insert boxes indicated areas of magnification shown below, and red arrowheads indicated areas of lanthanum nitrate deposition. Scale bar = 1 μm (upper) and 1 μm (bottom). **H** Representative immunofluorescence images of CD31 in cardiac tissue. Scale bar = 20 μm. **I** Quantitative analysis of blood vessel density in **H**. Data were presented as mean ± SEM. One-way ANOVA was used for statistical analysis

Adipsin^LSL/LSL^ and Adipsin^LSL/LSL^-Cre mice were randomly assigned to diabetic group (DM) and non-diabetic group (Non-DM). Following induction of experimental diabetes using the HFD/STZ approach, blood glucose levels were measured and revealed that Adipsin overexpression had no significant effect on blood glucose levels in diabetic group (Additional file 1: Table S4 and Additional file 2: Fig. S1A–C). The expression levels of Adipsin were detected using Western blot and quantitative real-time PCR in various adipose tissues (Additional file 2: Fig. S2A–C). Adipsin levels in serum, cardiac tissues and endothelial cells were significantly elevated in Adipsin^LSL/LSL^-Cre group as compared with Adipsin^LSL/LSL^ group (Fig. 2A–E).

It is also noteworthy that three-dimensional morphology of small blood vessels is difficult to assess using conventional histopathological techniques. Therefore, we performed a scanning electron microscopic analysis of microvascular corrosion casts (Fig. 2F). In diabetic mice, abnormal microvascular morphology was observed, accompanied by a large number of bumps and dips in the internal milieu of microvasculature. However, this injury was overtly alleviated in the Adipsin^LSL/LSL^-Cre group. Transmission electron microscopy revealed that Adipsin dampened leakage from the vessel lumen to the basal lamina after perfusion with lanthanum nitrate in diabetic mice (Fig. 2G). Additionally, immunofluorescent staining showed increased cardiac microvascular density in the Adipsin^LSL/LSL^-Cre group compared with Adipsin^LSL/LSL^ group (Fig. 2H, I). These results indicated that Adipsin overexpression was involved in the regulation of microvascular dysfunction in diabetic cardiomyopathy.

### Adipsin mitigates cardiac dysfunction in diabetic cardiomyopathy

**Fig. 3:**
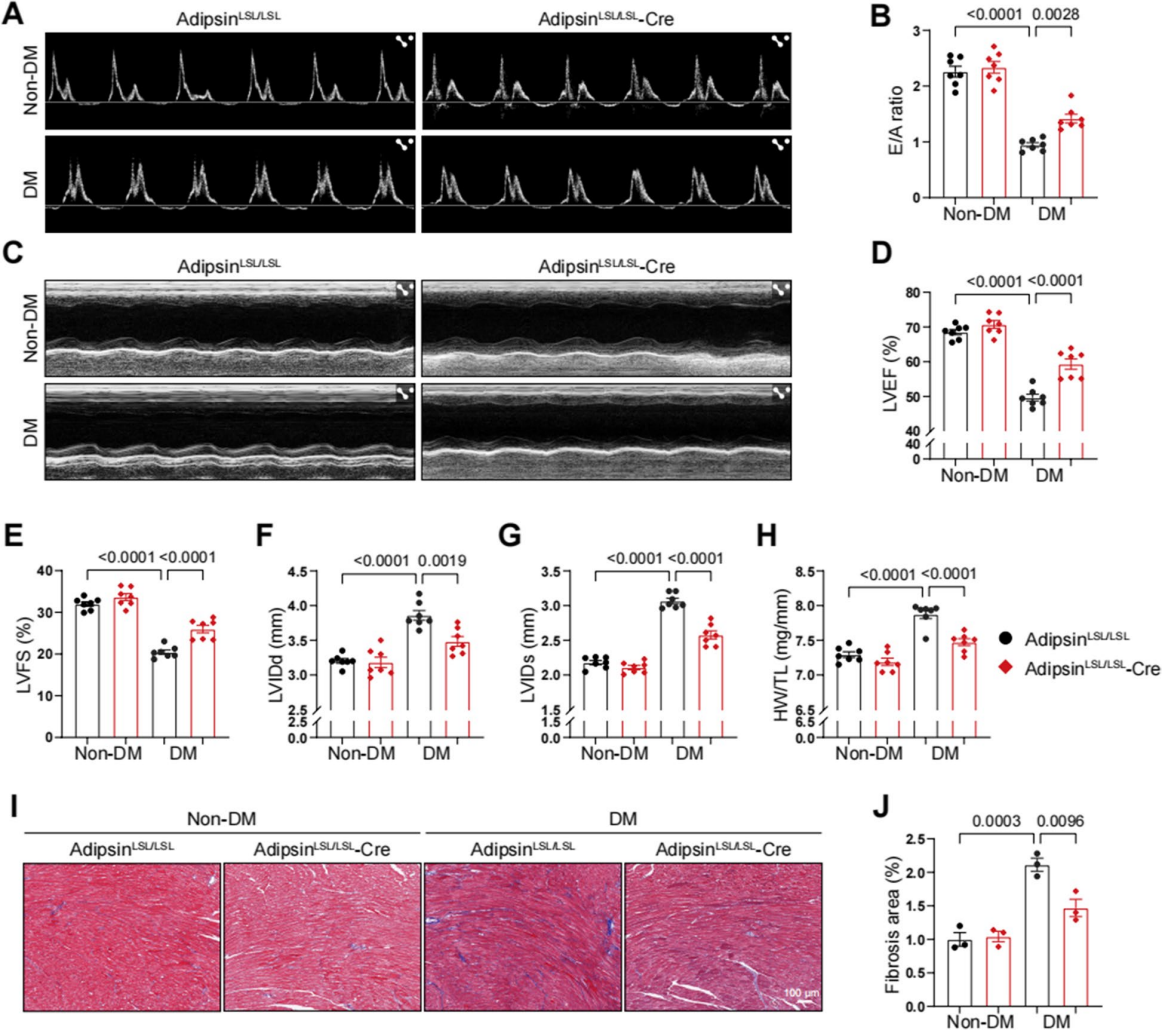
Adipose tissue-specific Adipsin overexpression inhibits cardiac hypertrophy and improves cardiac function. **A** Representative echocardiographic images of cardiac diastolic function. **B** Quantitative analysis of E/A ratio. **C** Representative echocardiographic images of cardiac systolic function. **D**–**G** Quantitative analysis of left ventricular ejection fraction (LVEF), left ventricular fractional shortening (LVFS), left ventricular systolic internal dimension (LVIDs), and left ventricular diastolic internal dimension (LVIDd). **H** Ratio of heart weight (mg) to tibia length (mm). **I** Masson trichrome staining of cardiac tissue. Scale bar = 100 μm. **J** Quantification of the Masson staining density in **I**. Data were presented as mean ± SEM. One-way ANOVA was used for statistical analysis

Our data revealed that Adipsin^LSL/LSL^-Cre + DM mice demonstrated a significant improvement in cardiac function, exemplified by increased early flow velocity (E) to peak atrial velocity (A) ratio (E/A, Adipsin^LSL/LSL^-Cre + DM *vs.* Adipsin^LSL/LSL^ + DM, 1.42 *vs.* 0.95) (Fig. 3A, B). Simultaneously, there was also a noticeable enhancement of left ventricular ejection fraction (LVEF, 59.32% *vs.* 49.65%) and left ventricular fractional shortening (LVFS, 25.97% *vs.* 20.46%), a significant decrease of left ventricular systolic internal dimension (LVIDs, 2.58 mm *vs.* 3.07 mm) and left ventricular diastolic internal dimension (LVIDd, 3.48 mm *vs.* 3.86 mm) (Fig. 3C–G). Moreover, morphological analysis revealed that Adipsin overexpression attenuated diabetes-induced cardiac hypertrophy (Fig. 3H). Additionally, we found that Adipsin overexpression reduced myocardial fibrosis as revealed by Masson trichrome staining (Fig. 3I, J). These findings suggested that Adipsin alleviated adverse cardiac remodeling in diabetic cardiomyopathy.

### Adipsin-enriched exosomes improve cardiac dysfunction in experimental DCM

**Fig. 4:**
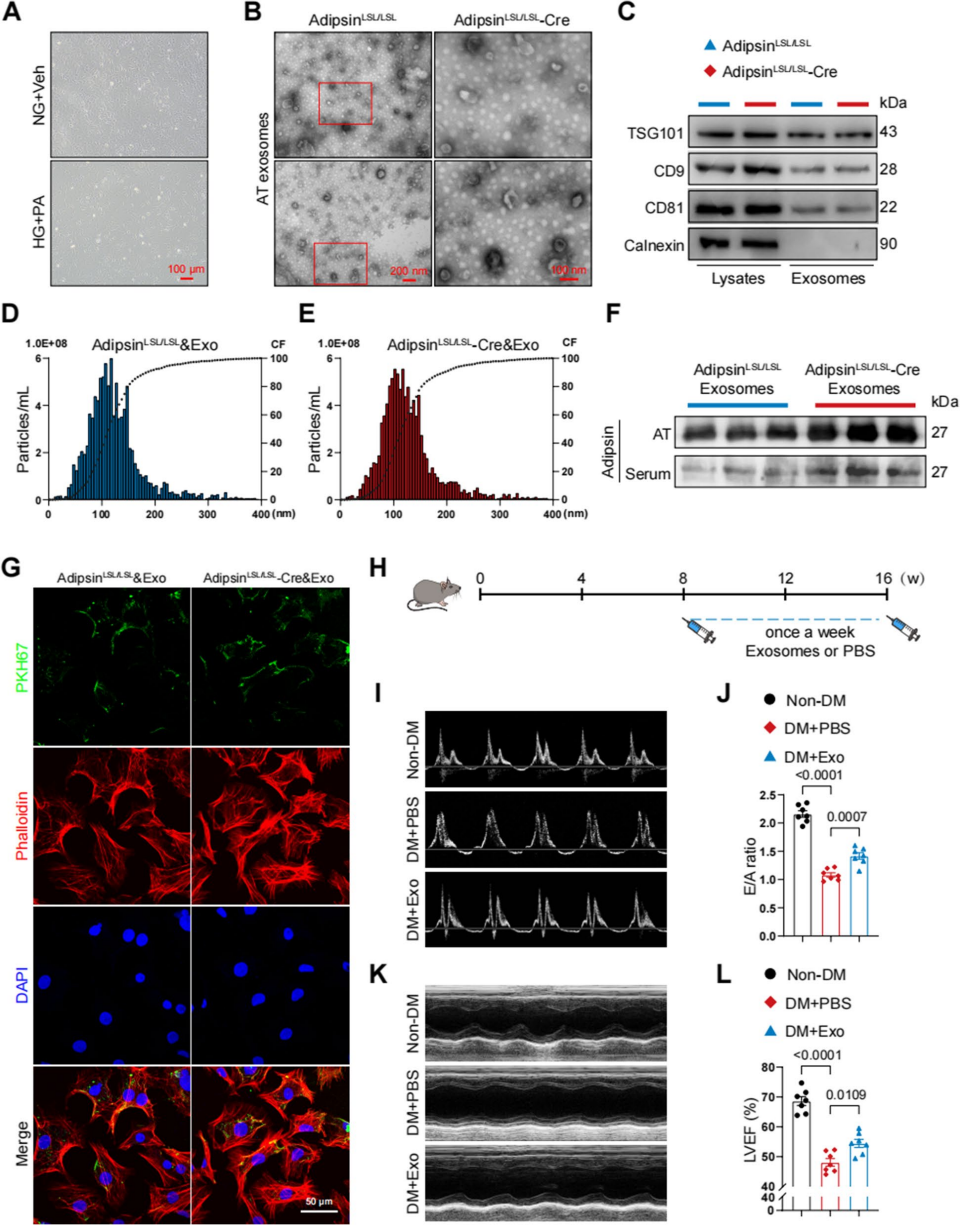
Exosomes secreted by adipose tissues can be taken up by CMECs and improve cardiac function in DCM. **A** Representative images of primary cardiac microvascular endothelial cells treated with NG + vehicle or HG + PA. Scale bar = 100 μm. **B** Representative transmission electron microscopy images of exosomes. Scale bar = 200 nm (left) and 100 nm (right). **C** Representative Western blot images of exosomes protein markers (positive: TSG101, CD9, and CD81; negative: Calnexin). **D**–**E** Representative size distribution of exosomes detected by nanoparticle tracking analysis. CF, cumulative frequencies. **F** Representative Western blot images of Adipsin levels in exosomes derived from serum and adipose tissue. **G** Representative immunofluorescence images of exosomes uptake. Scale bar = 50 μm. **H** Schematic illustration of experimental procedure. Mice were injected with exosomes isolated from adipose tissue of donor mice via tail veins. **I** Representative echocardiographic images of cardiac diastolic function. **J** Quantitative analysis of E/A ratio. **K** Representative echocardiographic images of cardiac systolic function. **L** Quantitative analysis of left ventricular ejection fraction (LVEF). Data were presented as mean ± SEM. One-way ANOVA was used for statistical analysis

To further validate Adipsin-offered protective effect in vitro, primary cardiac microvascular endothelial cells (CMECs) were isolated from mouse hearts and were subjected to acetylated low-density lipoprotein assay. CMECs were administered with high glucose and palmitic acid (HG + PA) to imitate glucotoxicity and lipotoxicity in vitro (Fig. 4A). Next, exosomes were isolated by culturing adipose tissue of Adipsin^LSL/LSL^ and Adipsin^LSL/LSL^-Cre mice in vitro. Transmission electron microscopy and nanoparticle tracking analyses were performed to identify exosomes (Fig. 4B–E). In addition, exosome marker proteins (TSG101, CD9, CD81, and Calnexin) were detected by Western blot (Fig. 4C). Adipsin levels were elevated in exosomes isolated from Adipsin^LSL/LSL^-Cre mice, compared with Adipsin^LSL/LSL^ mice (Fig. 4F and Additional file 2: Fig. S3A, B). Results showed that exosomes labeled with PKH67 were absorbed into CMECs (Fig. 4G). Intriguingly, Adipsin was barely detectable in serum after ultracentrifugation, which indicated that Adipsin was mainly encapsulated in extracellular vesicles (Additional file 2: Fig. S3C). We next evaluated the effect of Adipsin-enriched exosomes via tail vein injection on cardiac function in diabetic mice (Fig. 4H). Our findings indicated that Adipsin-enriched exosomes significantly ameliorated HFD/STZ-induced cardiac dysfunction as evidenced by increased E to A ratio and LVEF (Fig. 4I–L). Based on these results, possibilities were presented for the following study of adipose tissue exosomes exerting remote communication to improve cardiac dysfunction.

### Adipsin suppresses apoptosis and promotes proliferation in CMECs under diabetic condition

**Fig. 5:**
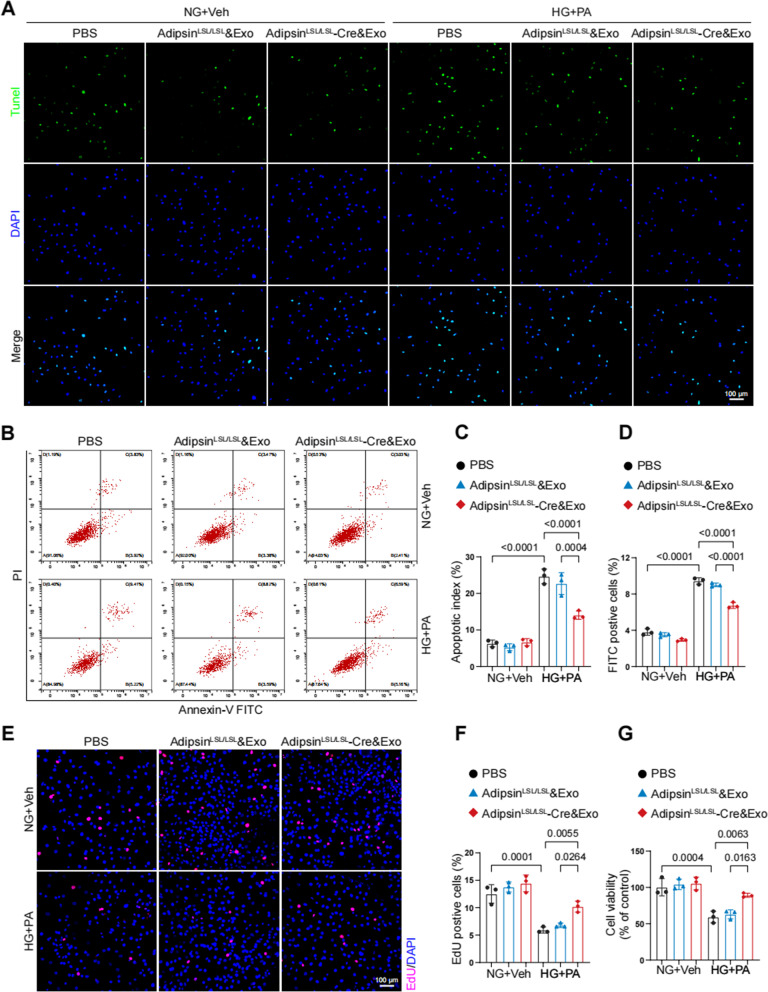
Adipsin promotes proliferation and suppresses apoptosis in CMECs under HG + PA conditions. **A** Representative images of apoptosis evaluated by TUNEL Assay (green cells) in vitro. Scale bar = 100 μm. **B** Representative dot plots displaying proportions of apoptotic cells. **D** Quantitative analysis of Annexin V-FITC-positive cells. **C** The apoptotic index was expressed as the number of TUNEL-positive cells normalized to the total number of cells. **E** Representative immunofluorescence images of CMEC proliferation evaluated by EdU-based proliferation assay. Scale bar = 100 μm. **F** Quantitative analysis of EdU-positive cells. **G** Cell viability determined using CCK-8 assay. Data were presented as mean ± SEM. One-way ANOVA was used for statistical analysis

CMECs were treated with exosomes secreted by different mouse adipose tissues (Adipsin^LSL/LSL^ and Adipsin^LSL/LSL^-Cre mice). HG + PA treatment increased CMECs apoptosis, while exosomes derived from Adipsin^LSL/LSL^-Cre mice suppressed apoptosis in CMECs (Fig. 5A). Evaluation of apoptotic index showed a noticeable decline in response to Adipsin-enriched exosomes. This observation was further confirmed by subsequent flow cytometry (Fig. 5B–D). A significant decrease of EdU-positive cells was observed in HG + PA condition. Interestingly, Adipsin treatment induced a noticeable rise of EdU-positive cells in Adipsin^LSL/LSL^-Cre&Exosomes group as compared with Adipsin^LSL/LSL^&Exosomes group (Fig. 5E, F). Meanwhile, CCK-8 assay showed that Adipsin significantly increased the proliferation ability of CMECs under diabetic condition (Fig. 5G). These results suggested that Adipsin inhibited apoptosis while increased proliferation after exposure to HG + PA in CMECs.

### Adipsin promotes cell migration during sprouting angiogenesis in CMECs

**Fig. 6:**
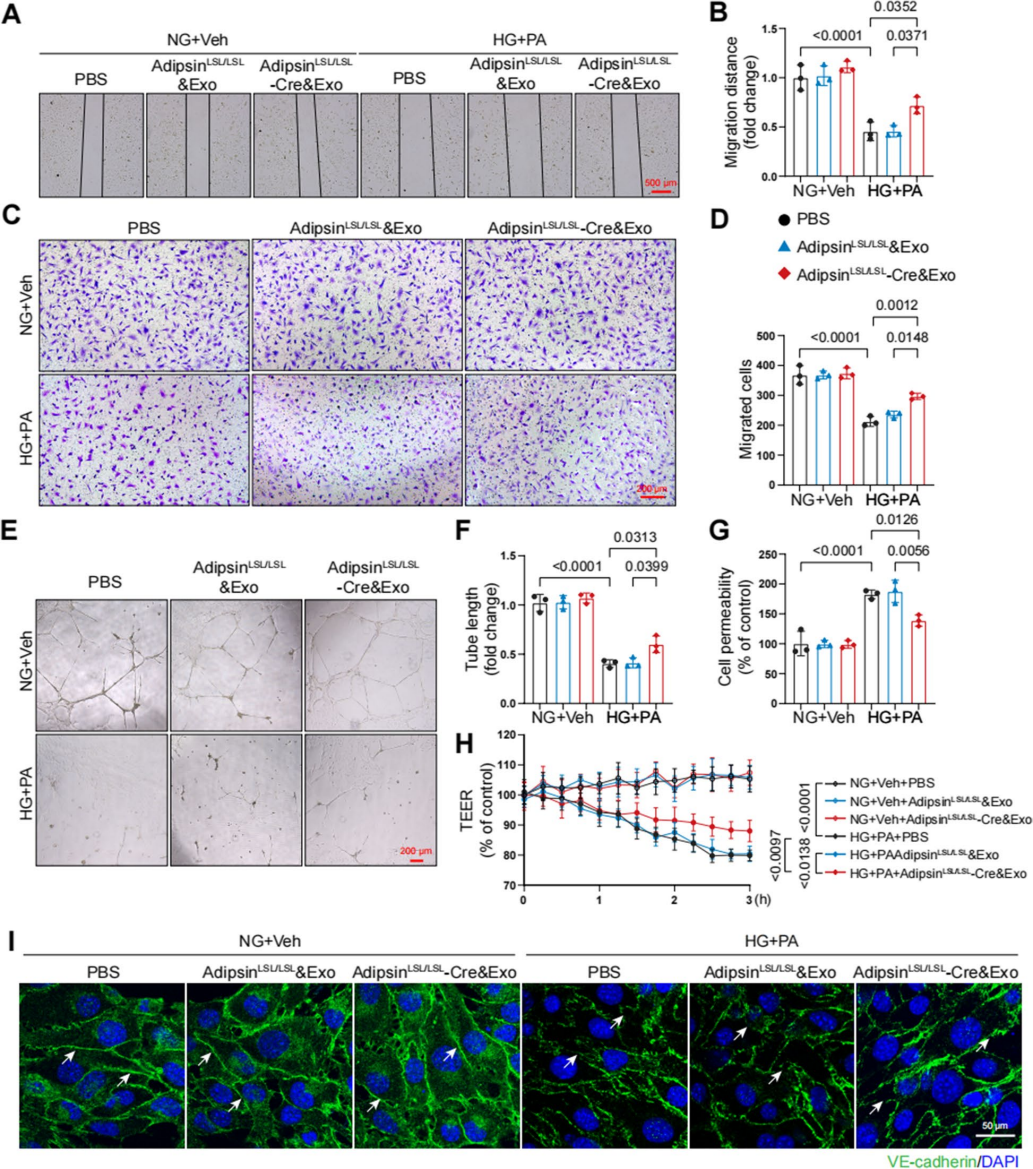
Adipsin promotes CMECs migration and tube formation while inhibits hyperpermeability in CMECs treated with HG + PA. **A** Wound healing scratches were imaged at 12 h after the initial scratch time point. Black lines indicated the boundary of the scratch. Scale bar = 500 μm. **B** Migration distances from edges of the wound. **C** Representative images of migrated cells in the transwell migration assay. Scale bar = 200 μm. **D** Quantitative analysis of migrated cells. **E** Representative images of tube formation assay. Scale bar = 200 μm. **F** Tube formation assays were quantified by measuring tube length. **G** Quantitative analysis of FITC-conjugated Dextran intensity evaluating monolayer cellular permeability. **H** Measurements of trans-endothelial electrical resistance (TEER). **I** Representative immunofluorescence images of cell-cell junctions, and white arrowheads indicated endothelial cell junctions. Scale bar = 50 μm. Data were presented as mean ± SEM. One-way ANOVA was used for statistical analysis

To investigate whether Adipsin affects endothelial function in vitro, we performed a series of experiments. The scratch-wound assay showed that speed of wound healing was accelerated in CMECs treated with Adipsin^LSL/LSL^-Cre&Exosomes, compared with Adipsin^LSL/LSL^&Exosomes (Fig. 6A, B). Transwell migration assay showed that Adipsin significantly rescued the HG + PA-mediated defect in cell migration (Fig. 6C, D). Additionally, endothelial tube formation assay showed that HG + PA suppressed tube formation, while Adipsin promoted tube formation under HG + PA insults (Fig. 6E, F). Additionally, Adipsin reversed permeability disrupted by HG + PA in monolayer endothelial cells as determined using the leakage of FITC-labeled dextran and trans-endothelial electrical resistance (TEER) analysis (Fig. 6G, H). Discontinuity of VE-cadherin distribution along the endothelial cell junctions was observed in HG + PA treatment group. Interestingly, Adipsin alleviated the disruption of endothelial cell junction integrity as indicated by improved continuity of VE-cadherin distribution along the endothelial cell junctions (Fig. 6I). These results confirmed the protective effects of Adipsin to regulate angiogenesis and permeability in CMECs.

### Adipsin maintains endothelial barrier integrity via inhibiting VE-cadherin internalization

**Fig. 7:**
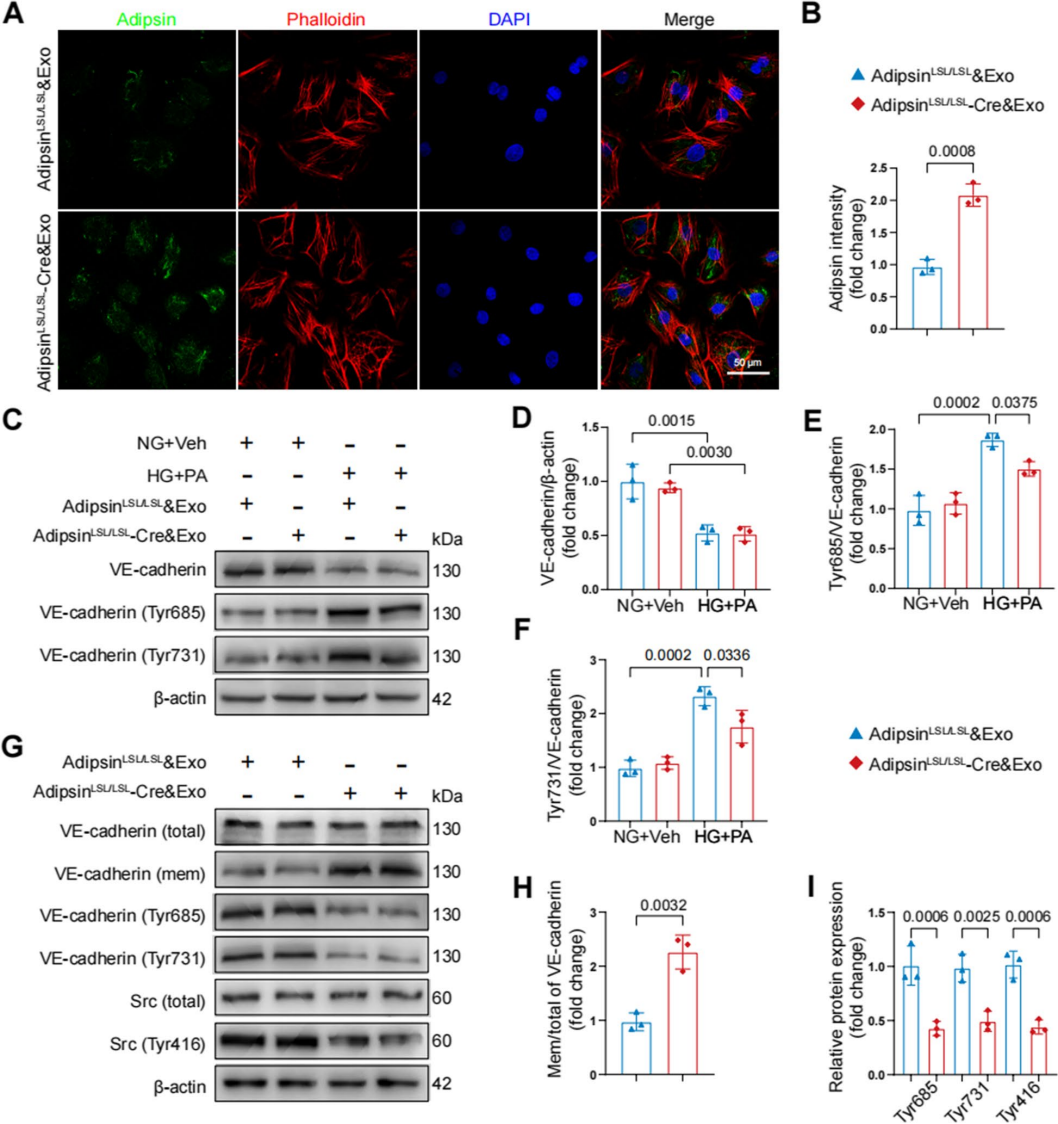
Adipsin interrupts HG + PA-induced VE-cadherin internalization by inhibiting Src activity. **A** Representative images of immunofluorescence staining for Adipsin in CMECs treated with Adipsin^LSL/LSL^&Exosomes or Adipsin^LSL/LSL^-Cre&Exosomes isolated from adipose tissues. Scale bar = 50 μm. **B** Quantitative analysis of Adipsin intensity in **A**. **C** Representative Western blot images for VE-cadherin (total), phospho-VE-cadherin (Tyr731), and phospho-VE-cadherin (Tyr685). **D**–**F** Quantitative analysis of Western blot images from three independent experiments in **C**. **G** Representative Western blot images for VE-cadherin (total), VE-cadherin (membrane), phospho-VE-cadherin (Tyr731), phospho-VE-cadherin (Tyr685), Src, and phospho-Src (Tyr416). **H**–**I** Quantitative Western blot analysis from three independent experiments in **G**. Data were presented as mean ± SEM. For **B**, **H**, and **I**, Student’s *t*‐test was used for statistical analysis. For **D**–**F**, one-way ANOVA was used for statistical analysis

To exploit the underlying mechanisms through which Adipsin regulates endothelial integrity, localization of Adipsin was evaluated in CMECs using immunofluorescence analysis. Adipsin displayed a predominantly cytoplasmic distribution (Fig. 7A, B). Next, the expression of cell junction molecules was not significantly altered in CMECs treated with Adipsin^LSL/LSL^&Exosomes or Adipsin^LSL/LSL^-Cre&Exosomes in HG + PA challenge (Additional file 2: Fig. S4). Western blot showed that HG + PA triggered VE-cadherin phosphorylation, while VE-cadherin phosphorylation was decreased in Adipsin^LSL/LSL^-Cre&Exosomes group (Fig. 7C, D). Western blot showed upregulation of membrane VE-cadherin, consistent with the reduction of VE-cadherin phosphorylation in Adipsin^LSL/LSL^-Cre&Exosomes group, compared with Adipsin^LSL/LSL^&Exosomes group (Fig. 7E, F). Src (proto-oncogene tyrosine-protein kinase Src) is well-known for its regulatory effects on VE-cadherin phosphorylation [28]. As shown, HG + PA increased, while Adipsin inhibited Src phosphorylation (Fig. 7G–I). These findings demonstrated that Adipsin blocked HG + PA-induced Src phosphorylation (Tyr416), VE-cadherin phosphorylation (Tyr685 and Tyr731) and VE-cadherin internalization, thus maintaining endothelial barrier integrity.

### Adipsin inhibits the phosphorylation of Src via binding to Csk

**Fig. 8:**
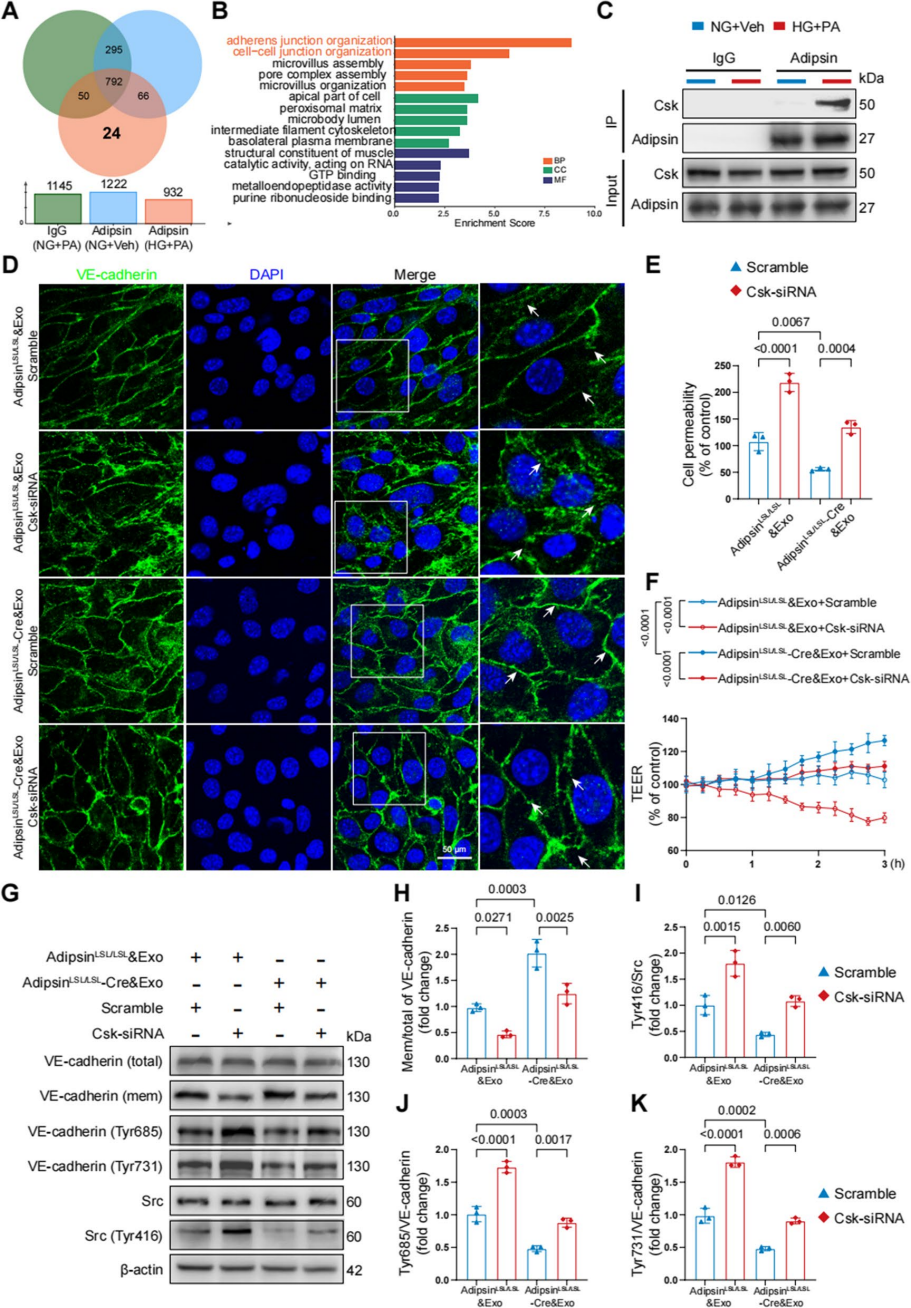
Adipsin directly bounds to Csk, impeding Src activity and VE-cadherin internalization. **A** Venn diagram showed the protein candidates as the targets of Adipsin. **B** GO analysis of Adipisn-associated proteins. GO, Gene Ontology; BP, biological process; CC, cellular component; MF, molecular function. **C** Representative Western blot images for co-immunoprecipitation of Adipsin interacting with Csk. **D** Representative immunofluorescence images of cell-cell junctions, and white arrowheads indicated endothelial cell junctions. Scale bar = 50 μm. **E** Quantitative analysis of FITC-conjugated Dextran intensity evaluating monolayer cellular permeability. **F** Measurements of trans-endothelial electrical resistance (TEER). **G** Representative Western blot images for VE-cadherin (total), VE-cadherin (membrane), phospho-VE-cadherin (Tyr685), phospho-VE-cadherin (Tyr731), Src, and phospho-Src (Tyr416). **H**–**K** Quantitative analysis of Western blot images from three independent experiments in **G**. Data were presented as mean ± SEM. One-way ANOVA was used for statistical analysis

To elucidate how Adipsin suppresses HG + PA-induced VE-cadherin internalization, CMECs were treated with Adipsin^LSL/LSL^&Exosomes or Adipsin^LSL/LSL^-Cre&Exosomes to screen potential target of Adipsin using LC-MS/MS analysis (Additional file 1: Table S5). A list of candidate proteins was generated from GO enrichment analysis (Fig. 8A, B). Among these proteins, Csk (tyrosine-protein kinase) was noted since Csk is a negative regulator of Src activation [29]. Interestingly, Adipsin did not affect Csk mRNA and protein expression in CMECs exposed to HG + PA treatment (Additional file 2: Fig. S5). Based on the aforementioned analysis, Csk was identified as a downstream signaling molecule for further interest.

To further validate possible interaction between Adipsin and Csk, co-immunoprecipitation analysis (Co-IP) was performed. As expected, there was a direct interaction between Adipsin and Csk (Fig. 8C). Next, we performed loss-of-function studies to corroborate Csk as a downstream regulator of Adipsin. Immunofluorescence staining was performed, and results indicated that Csk knockdown counteracted Adipsin-mediated evident increase of membrane VE-cadherin on disrupted endothelial cell junctions (Fig. 8D). FITC-labeled dextran analysis and TEER also demonstrated that Csk knockdown abolished Adipsin-offered protective effects on CMECs leakage (Fig. 8E, F). Furthermore, Csk knockdown abolished the inhibition of VE-cadherin internalization exerted by Adipsin (Fig. 8G–K). These findings suggested that Adipsin could bind to Csk, thus suppressing Src phosphorylation and VE-cadherin phosphorylation, which prevented VE-cadherin internalization.

### Csk deficiency abrogates microvascular protective effects of Adipsin

**Fig. 9:**
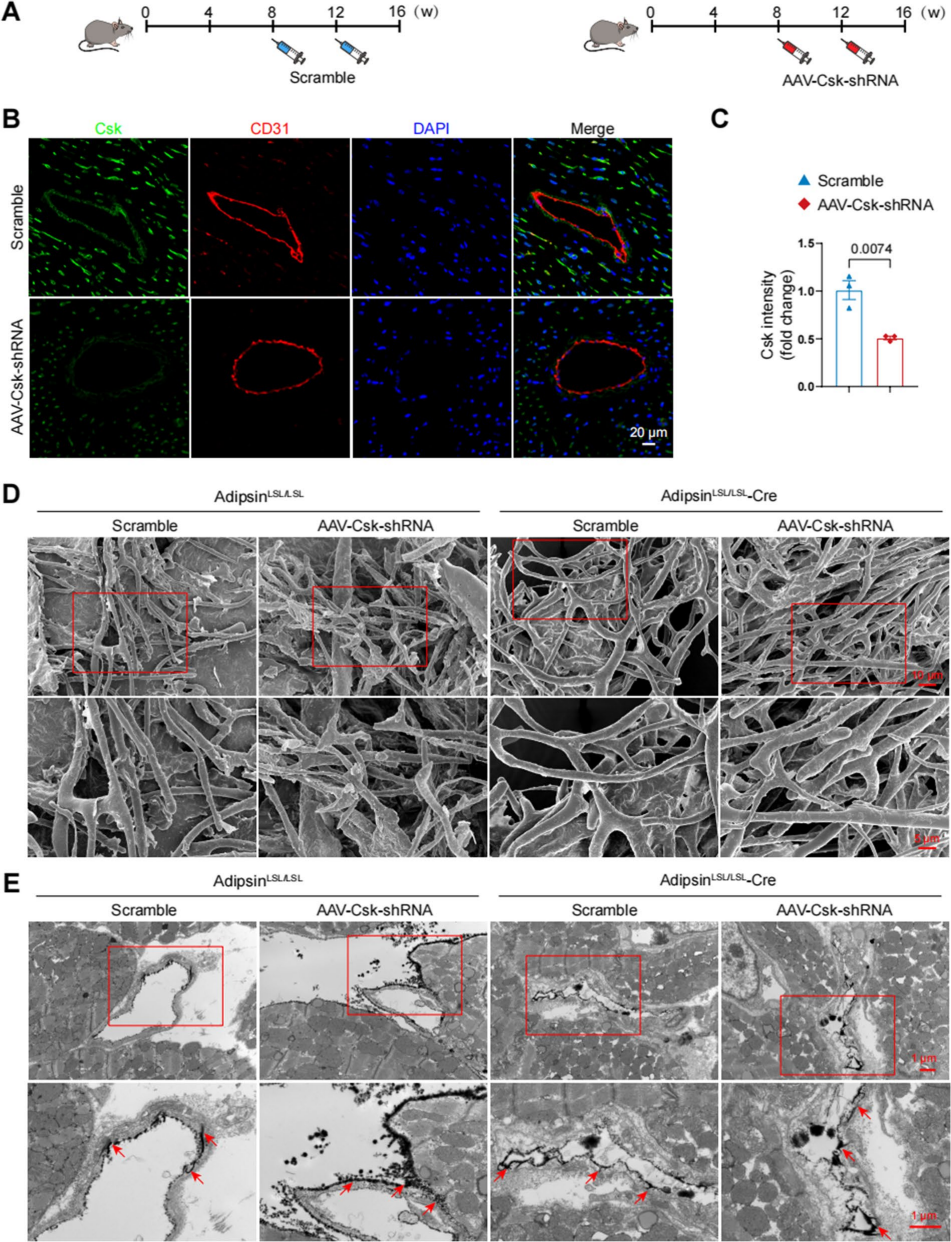
Csk serves as a critical downstream target of Adipsin. **A** Schematic illustration of adeno-associated virus 9 delivery leading to knockdown of Csk in CMECs. **B** Representative immunofluorescence images of Csk in cardiac tissue. Scale bar = 20 μm. **C** Quantitative analysis of Csk fluorescence intensity in **B**. **D** Representative scanning electron micrographs of cardiac microvessels corrosion in different groups. Inserted boxes indicated areas of magnification shown below. Scale bar = 10 μm (upper) and 5 μm (bottom). **E** Representative transmission electron micrographs of microvascular leakage of lanthanum nitrate evaluating endothelial permeability. Inserted boxes indicated areas of magnification shown below, and red arrowheads indicated areas of lanthanum nitrate deposition. Scale bar = 1 μm (upper) and 1 μm (bottom). Data were presented as mean ± SEM. Student’s *t*‐test was used for statistical analysis

To further discern the effect of Adipsin-enriched exosomes on Csk/Src/VE-cadherin signaling pathways, an adeno-associated virus 9 (AAV9) vector was employed to downregulate the expression of Csk in vivo. Adipsin^LSL/LSL^ and Adipsin^LSL/LSL^-Cre mice were randomly injected with AAV9-Csk-shRNA or scramble following the final STZ injection in cardiac tissues (Fig. 9A). To validate the effectiveness of Csk shRNA, we utilized immunofluorescent double-labeling to determine Csk expression in endothelial cells (Fig. 9B, C). Cardiac microvascular corrosion casts showed that Adipsin overexpression ameliorated HG + PA-induced endothelial barrier integrity destruction, while AAV9-Csk-shRNA treatment abolished these effects (Fig. 9D). Extravasation of lanthanum nitrate analysis demonstrated an enhanced microvascular permeability in the AAV9-Csk-shRNA group compared to the scramble group in diabetic mice (Fig. 9E). Based on these results, Csk appeared to play a critical role in Adipsin-evoked protective effects on cardiac microvascular injury.

## Discussion

The ever-rising prevalence of diabetes and multiple organ complications have imposed a major public health threat. Microvascular complications account for a 40% additional probability of experiencing a major adverse cardiovascular event in patients with diabetes [3]. Effective management of microvascular complications offers intense potential to improve the prognosis of diabetic patients. The major findings from our current study found that serum Adipsin levels were remarkably declined in type 2 diabetic patients and animal models of type 2 diabetes. Adipose tissue-specific Adipsin overexpression alleviated coronary microvascular injury, enhanced cardiac diastolic and systolic function. These data indicated that Adipsin may serve as a treatment target against diabetic coronary microvascular injury and subsequent diabetic cardiomyopathy.

Diabetic patients exhibit diffuse coronary artery negative remodeling and stenosis. This scenario is associated with an increased prevalence of myocardial infarction and cardiac death. In fact, diabetic microangiopathy and macroangiopathy are not completely distinct entities. They should be viewed as a continuum of the widespread vascular damage induced by diabetes. Our previous study demonstrated that Adipsin inhibited lipid uptake in a PPARγ/CD36-dependent manner, thus preventing the formation of foam cells, and stabilizing atherosclerotic plaque [30]. Adipsin also inhibited cardiomyocyte ferroptosis, maintained iron homeostasis and alleviated cardiac dysfunction in MI injury [31]. These notions have thus raised a possibility that Adipsin might participate in the pathogenesis of coronary microvascular injury in the face of diabetic insult. In this study, scanning electron microscope indicated obvious bumps and dips in the internal milieu of the microvasculature. Cardiac microvascular permeability was increased in response to diabetic insults. Interestingly, adipose tissue-specific Adipsin overexpression significantly reduced cardiac microvascular permeability, maintained coronary microvascular integrity, and increased coronary microvascular density. These results indicated the role of Adipsin as a potential therapeutic target in diabetic coronary microvascular injury. Ample evidence has indicated the occurrence of diabetic cardiomyopathy by early-stage microvascular injury and end-stage adverse cardiac remodeling [23, 24]. Of interest, Adipsin overexpression also attenuated adverse cardiac remodeling and cardiac dysfunction in diabetic mice. These findings have provided insights into the protective effects of Adipsin in diabetic cardiomyopathy.

Extracellular vesicles (EVs), including exosomes, microvesicles (MVs), and apoptotic bodies, are characterized by membranous subcellular structures with lipid bilayers and cytoplasmic components [32, 33]. EVs derived from adipose tissues have been implicated in various metabolic diseases, including diabetes and insulin resistance [34–36]. The role of EVs in the pathogenesis of diabetic complications has been attracting ever-rising attention in the past few years [37, 38]. Kumar and coworkers reported the involvement of fecal exosomes in the development of insulin resistance in mice fed a high-fat diet [39]. Our present study indicated that adipose tissues released Adipsin-enriched exosomes, the smallest EVs with a size range of 50–150 nm. Adipsin-enriched exosomes can be taken up by CMECs, thus suppressing CMECs apoptosis and facilitating CMECs proliferation, and relieving HFD/STZ-induced cardiac dysfunction. These results provided direct evidence that Adipsin-enriched exosomes mediate remote communication between adipose tissue and heart which mitigated coronary microvascular injury in diabetic mice.

The monolayer of CMECs forms a semipermeable barrier between circulating substances and cardiac myocardium. Cell junctions between CMECs serve as the main structures to sustain coronary microvascular integrity and cardiac microenvironment homeostasis. Hence, elucidating signaling mechanisms responsible for the regulation of CMECs junctions is of paramount importance to the development of novel therapeutic strategies for diabetic coronary microvascular injury. VE-cadherin is pivotal in the assembly and maintenance of adherens junctions (AJs) [40, 41]. Multiple lines of evidence have demonstrated that VE-cadherin cellular internalization disrupted the VE-cadherin adhesion complex [42–45]. This study favored that VE-cadherin phosphorylation and VE-cadherin internalization were increased in response to diabetes insult. Adipsin-enriched exosomes inhibited VE-cadherin phosphorylation, reversed VE-cadherin internalization, thus maintained the integrity of CMECs adherens junctions.

Vessel permeability function is associated with Src-dependent phosphorylation of VE-cadherin [46]. Wang and associates noted involvement of Src/VE-cadherin signaling pathway in pulmonary endothelial barrier dysfunction [47]. VEGF/Src signaling cascade was found to be implicated in the regulation of VE-cadherin expression in experimental diabetic retinopathy [48]. In this study, Adipsin-enriched exosomes suppressed Src phosphorylation and stabilized VE-cadherin adhesion in response to diabetes-induced microvascular hyperpermeability. Bioinformatic analysis revealed 24 proteins that are capable of directly interacting with Adipsin. Of interest, several studies have pinpointed the role of Csk as a negative regulator for Src [29, 49]. Our findings demonstrated a direct interaction between Adipsin and Csk. Interestingly, Csk knockdown abolished the effects of Adipsin on CMECs leakage. The effects of Adipsin on VE-cadherin phosphorylation and internalization were also counteracted by Csk knockdown. In vivo study revealed that Csk knockdown abolished the effects of Adipsin on endothelial barrier integrity and microvascular permeability. These results indicated that Csk is a downstream regulator of Adipsin-mediated protection against microvascular hyperpermeability.

## Conclusions

These lines of evidence suggest that Adipsin mitigates diabetes-associated coronary microvascular complications. Adipsin directly interacts with Csk, blocks VE-cadherin internalization, and stabilizes adherens junction, thus mitigates CMECs hyperpermeability induced by diabetes insult. Adipsin may serve as a novel target for the treatment of coronary microvascular injury in diabetic patients.

## Supplementary Information

**Additional file 1: Figure S1.** Schematic diagram of transgenic mice; **Figure S2.** Construction of Adeno-associated Virus 9; **Table 1.** Antibody information and dilutions; **Table 2.** Primer sequences; **Table 3.** General characteristics of healthy individuals and type 2 diabetic patients; **Table 4.** Blood glucose in mice; **Table 5.** Mass spectrometry protein analysis.

**Additional file 2: Figure S1.** Changes in blood glucose levels. **Figure S2.** Changes in Adipsin levels. **Figure S3.** Adipsin levels in exosomes. **Figure S4.** Expression levels of cell junction molecules are unaffected in CMECs treated with Adipsin^LSL/LSL^&Exosomes or Adipsin^LSL/LSL^-Cre&Exosomes under HG + PA challenge. **Figure S5.** Adipsin^LSL/LSL^-Cre&Exosomes administration has no effects on Csk expression in CMECs.

**Additional file 3.** Original data of Western blots imaging.

## Data Availability

All data generated or analyzed during this study are included in this published article and its supplementary information files or from the corresponding author upon reasonable request.

## Abbreviations

AAV9: Adeno-associated virus 9
BAT: Brown adipose tissue
CMECs: Cardiac microvascular endothelial cells
Csk: Tyrosine-protein kinase Csk
DCM: Diabetic cardiomyopathy
EdU: 5-Ethynyl-2′-deoxyuridine
eWAT: Epididymal white adipose tissue
GO: Gene ontology
HFD: High-fat diet
HG: High glucose
iWAT: Inguinal white adipose tissue
PA: Palmitic acid
shRNA: Short hairpin RNA
Src: Proto-oncogene tyrosine-protein kinase Src
STZ: Streptozotocin
T2DM: Type 2 diabetes mellitus
TEER: Trans-endothelial electrical resistance
