# Supplementary material for: Adipsin alleviates cardiac microvascular injury in diabetic cardiomyopathy through Csk-dependent signaling mechanism
Source: BMC Med. 2023 May 26;21:197. doi: 10.1186/s12916-023-02887-7 (PMC10224320; doi:10.1186/s12916-023-02887-7)
Supplement: Supplementary file 1 — Additional file 1: Figure S1. Schematic diagram of transgenic mice; Figure S2. Construction of Adeno-associated Virus 9; Table 1. Antibody information and dilutions; Table 2. Primer sequences; Table 3. General characteristics of healthy individuals and type 2 diabetic patients; Table 4. Blood glucose in mice; Table 5. Mass spectrometry protein analysis. [file 12916_2023_2887_MOESM1_ESM.docx]

**Additional File 1**

**Figure S1 Schematic diagram of transgenic mice**

Gene: Gt(ROSA)26Sor (ENSMUSG00000086429)

http://asia.ensembl.org/Mus_musculus/Gene/Summary?db=core;g=ENSMUSG00000086429;r=6:113067428-113077333

Sites: Chromosome 6: 113,076,031 (Ensembl)

**Adipsin^LSL/+^ mice**

**
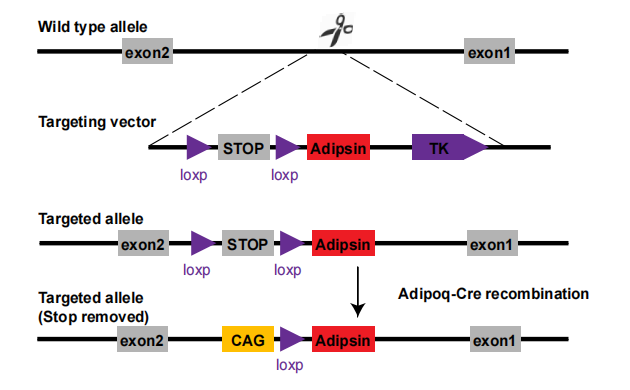
**

**Adipoq-Cre mice**

Gene: transgene insertion 1, Evan Rosen

Mice harboring the Adipoq-Cre BAC transgene express Cre recombinase under control of the mouse adiponectin (Adipoq) promoter/enhancer regions within the BAC transgene. The transgene integration site was identified to be on chromosome 9 between exons 6 and 7 of the *Tbx18* locus.

**Figure S2 Construction of Adeno-associated Virus 9 (AAV9)**

**
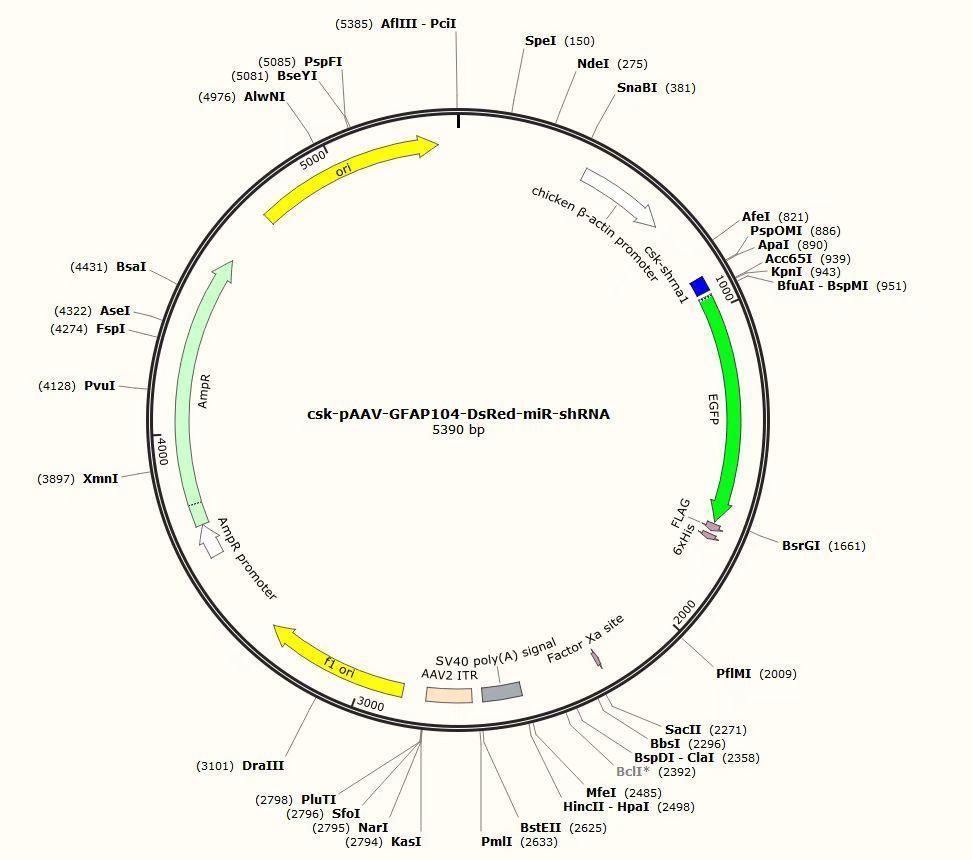
**

**Csk-shRNA:**

GAAGCGCACAGACAAAGGCTCGAGCCTTTGTCTGTGCGCTTCTTTTT

**Table S1 Antibody information and dilutions**

| **Genes** | **Manufacturer** | **Number** | **Application** |
| --- | --- | --- | --- |
| **Adipsin** | Abcam | ab204917 | WB 1:5000 |
| **Csk** | Abcam | ab244414 | WB 1:1000  IF 1:100 |
| **Transferrin** | Abcam | ab82411 | WB 1:10000 |
| **Pho-VE-cadherin (Tyr685)** | Abcam | ab119785 | WB 1:1000 |
| **Adipsin** | ABclonal | A8117 | WB 1:1000 |
| **Calnexin** | ABclonal | A15631 | WB 1:1000 |
| **VE-cadherin** | Thermo Fisher Scientific | 36-1900 | WB 1:2000  IF 1:100 |
| **Src** | Thermo Fisher Scientific | 44-656G | WB 1:1000 |
| **Phos-Src**  **(Tyr416)** | Thermo Fisher Scientific | PA5-97364 | WB 1:1000 |
| **Connexin-43** | Proteintech | 26980-1-AP | WB 1:4000 |
| **JAM-A** | Proteintech | 16183-1-AP | WB 1:1000 |
| **TSG101** | Proteintech | 67381-1-Ig | WB 1:5000 |
| **Calnexin** | Proteintech | 10427-2-AP | WB 1:5000 |
| **CD81** | Proteintech | 66866-1-Ig | WB 1:1000 |
| **CD9** | Proteintech | 20597-1-AP | WB 1:1000 |
| **Gapdh** | Proteintech | 10494-1-AP | WB 1:5000 |
| **β-actin** | Proteintech | 20536-1-AP | WB 1:5000 |
| **β-catenin** | Proteintech | 66379-1-Ig | WB 1:5000 |
| **Adipsin** | Santa Cruz Biotechnology | sc-376015 | WB 1:1000  IP 1:50  IF 1:50 |
| **ZO-1** | Servicebio | GB111402 | WB 1:1000 |
| **CD31** | Servicebio | GB12063 | IF 1:100 |
| **Occludin** | Cell Signaling Technology | 91131S | WB 1:1000 |
| **Claudin-5** | Cell Signaling Technology | 49564 | WB 1:1000 |
| **Pho-VE-cadherin (Tyr731)** | Sigma-Aldrich | SAB4504676 | WB 1:1000 |

**Table S2 Primer sequences**

| **Genes** | **Forward primer (5'—3')** | **Reverse primer (5'—3')** |
| --- | --- | --- |
| **Adipsin** | GCAGCACAGCCCCGAG | GTCGTCATCCGTCACTCCAT |
| **β-actin** | AACAGTCCGCCTAGAAGCAC | CGTTGACATCCGTAAAGACC |
| **VE-cadherin** | CACTGCTTTGGGAGCCTTC | GGGGCAGCGATTCATTTTTCT |
| **β-catenin** | ATGGAGCCGGACAGAAAAGC | CTTGCCACTCAGGGAAGGA |
| **ZO-1** | GCCGCTAAGAGCACAGCAA | TCCCCACTCTGAAAATGAGGA |
| **Occludin** | TTGAAAGTCCACCTCCTTACAGA | CCGGATAAAAAGAGTACGCTGG |
| **Connexin-43** | ACAGCGGTTGAGTCAGCTTG | GAGAGATGGGGAAGGACTTGT |
| **JAM-A** | TCTCTTCACGTCTATGATCCTGG | TTTGATGGACTCGTTCTCGGG |
| **Claudin-5** | GCAAGGTGTATGAATCTGTGCT | GTCAAGGTAACAAAGAGTGCCA |
| **Csk** | TTCCCTTCTGCAAAGGAGATGT | ACCAGGGCATAAGGCTGAGT |

**Table S3 General characteristics of healthy individuals and type 2 diabetic patients.**

| **Clinical Characteristic** | **Healthy Individuals(n=30)** | **T2DM(n=30)** |
| --- | --- | --- |
| **Male**  **n(%)** | 23  (76.7) | 24  (80.0) |
| **Age**  **(years)** | 64.10±10.23 | 64.30±7.92 |
| **Body mass index**  **(kg/m**2) | 24.16±2.20 | 24.15±2.55 |
| **Total cholesterol**  **(mmol/L)** | 3.31±0.72 | 3.47±1.13 |
| **Triglyceride**  **(mmol/L)** | 1.32±0.56 | 1.63±1.01 |
| **High-density lipoprotein**  **(mmol/L)** | 1.11±0.27 | 1.04±0.24 |
| **Low-density lipoprotein**  **(mmol/L)** | 1.67±0.48 | 1.91±1.06 |
| **Systolic blood pressure**  **mmHg** | 123.60±12.37 | 126.70±22.89 |
| **Diastolic blood pressure**  **mmHg** | 68.83±6.48 | 70.20±11.11 |
| **HbA1c**  **(%)** | 5.27±1.20 | 8.64±1.45* |
| **Fasting blood glucose**  **(mmol/L)** | 5.19±1.03 | 10.09±0.96* |
| **Serum Adipsin**  **(ng/ml)** | 2588.0±534.0 | 1538.0±323.0* |

Data were presented as mean ± SD. **p* < 0.05 vs. Healthy Individuals.

**Table S4 Blood glucose in mice**

| **Blood glucose**  **(mmol/L)** | **Non-DM** | | **DM** | |
| --- | --- | --- | --- | --- |
|  | **Adipsin^LSL/LSL^** | **Adipsin^LSL/LSL^-Cre** | **Adipsin^LSL/LSL^** | **Adipsin^LSL/LSL^-Cre** |
| **0** | 9.10±1.33 | 9.31±1.50 | 9.10±1.89 | 8.80±1.40 |
| **4** | 10.16±1.99 | 9.94±1.43 | 10.53±1.75 | 9.83±3.31 |
| **8** | 8.89±1.61 | 8.60±1.68 | 19.86±2.08* | 19.64±1.40^#^ |
| **12** | 9.79±1.94 | 9.70±1.29 | 24.77±4.36* | 23.16±3.60^#^ |
| **16** | 9.33±2.53 | 10.40±1.55 | 23.37±4.16* | 25.60±4.28^#^ |

Data were presented as mean ± SEM. **p* < 0.05 vs. Adipsin^LSL/LSL^ + Non-DM group. #*p* < 0.05 vs. Adipsin^LSL/LSL^-Cre + Non-DM group.

**Table S5 Mass spectrometry protein analysis**

| Protein | Protein Name | MolWeight | LFQ intensity |
| --- | --- | --- | --- |
| Q8BQS4 | Protein FAM102B | 36.513 | 354210 |
| **P41241** | **Tyrosine-protein kinase CSK** | **50.716** | **116357.5** |
| Q9WTX8 | Mitotic spindle assembly checkpoint protein MAD1 | 83.54 | 103420 |
| Q9QXS1 | Plectin | 534.18 | 94135 |
| P05977 | Myosin light chain 1/3, skeletal muscle isoform | 20.594 | 86651 |
| O35598 | Disintegrin and metalloproteinase domain-containing protein 10 | 83.967 | 48161 |
| E9Q557 | Desmoplakin | 332.91 | 42438.2 |
| Q9JHR7 | Insulin-degrading enzyme | 117.77 | 24283.5 |
| Q9QXB9 | Developmentally-regulated GTP-binding protein 2 | 40.718 | 22569 |
| P26043 | Radixin | 68.542 | 14753.1 |
| P42932 | T-complex protein 1 subunit theta | 59.555 | 11382.5 |
| P12710 | Fatty acid-binding protein, liver | 14.245 | 10878.5 |
| Q6IRU2 | Tropomyosin alpha-4 chain | 28.467 | 10342.5 |
| Q8K268 | ATP-binding cassette sub-family F member 3 | 79.864 | 8838 |
| O55236 | mRNA-capping enzyme | 68.683 | 7532 |
| P60766 | Cell division control protein 42 homolog | 21.258 | 7282 |
| Q8BWQ4 | Cap-specific mRNA (nucleoside-2'-O-)-methyltransferase 2 | 87.142 | 6615.5 |
| Q9Z280 | Phospholipase D1 | 123.97 | 6400.5 |
| O35730 | E3 ubiquitin-protein ligase RING1 | 42.63 | 5533 |
| Q9D2C6 | DNA-directed RNA polymerase III subunit RPC8 | 22.948 | 3061.6 |
| Q9CYC6 | m7GpppN-mRNA hydrolase | 48.379 | 2302.35 |
| P99027 | 60S acidic ribosomal protein P2 | 11.651 | 2141.95 |
| Q8BRH4 | Histone-lysine N-methyltransferase 2C | 540.18 | 1751.2 |
| Q80WQ2 | Protein VAC14 homolog | 88.047 | 749.5 |
