# Supplementary material for: Adipsin alleviates cardiac microvascular injury in diabetic cardiomyopathy through Csk-dependent signaling mechanism
Source: BMC Med. 2023 May 26;21:197. doi: 10.1186/s12916-023-02887-7 (PMC10224320; doi:10.1186/s12916-023-02887-7)
Supplement: Supplementary file 2 — Additional file 2: Figure S1. Changes in blood glucose levels. Figure S2. Changes in Adipsin levels. Figure S3. Adipsin levels in exosomes. Figure S4. Expression levels of cell junction molecules are unaffected in CMECs treated with AdipsinLSL/LSL&Exosomes or AdipsinLSL/LSL-Cre&Exosomes under HG + PA challenge. Figure S5. AdipsinLSL/LSL-Cre&Exosomes administration has no effects on Csk expression in CMECs. [file 12916_2023_2887_MOESM2_ESM.docx]

**Additional File 2**

**Figure S1**


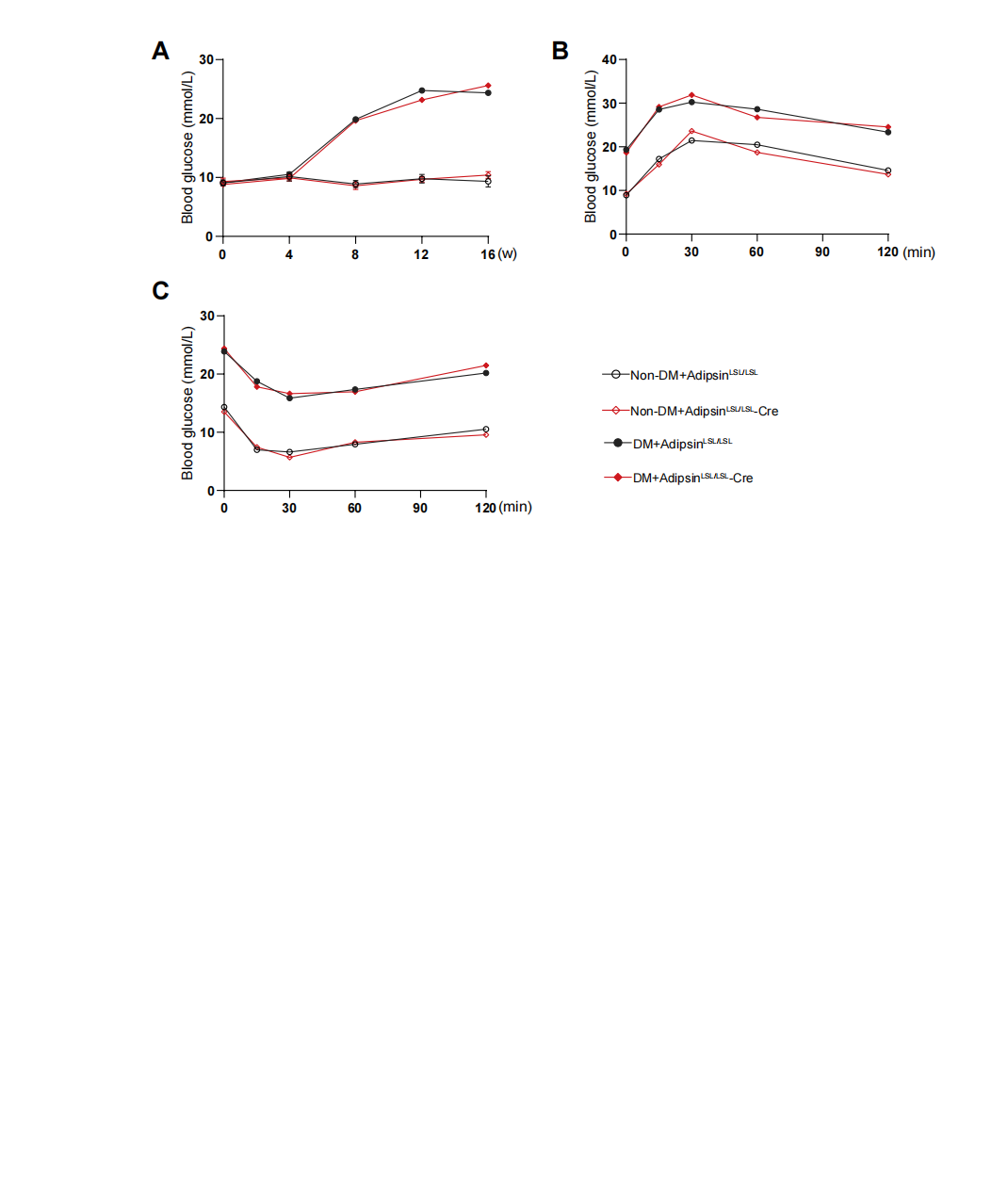


**Figure S1 Changes in blood glucose levels. (A)** Changes in blood glucose levels at 0 weeks, 4 weeks, 8 weeks, 12 weeks, and 16 weeks following high-fat diet feeding. (B) Blood glucose levels of the glucose tolerance test. (C) The glucose levels of the insulin tolerance test.

**Figure S2**


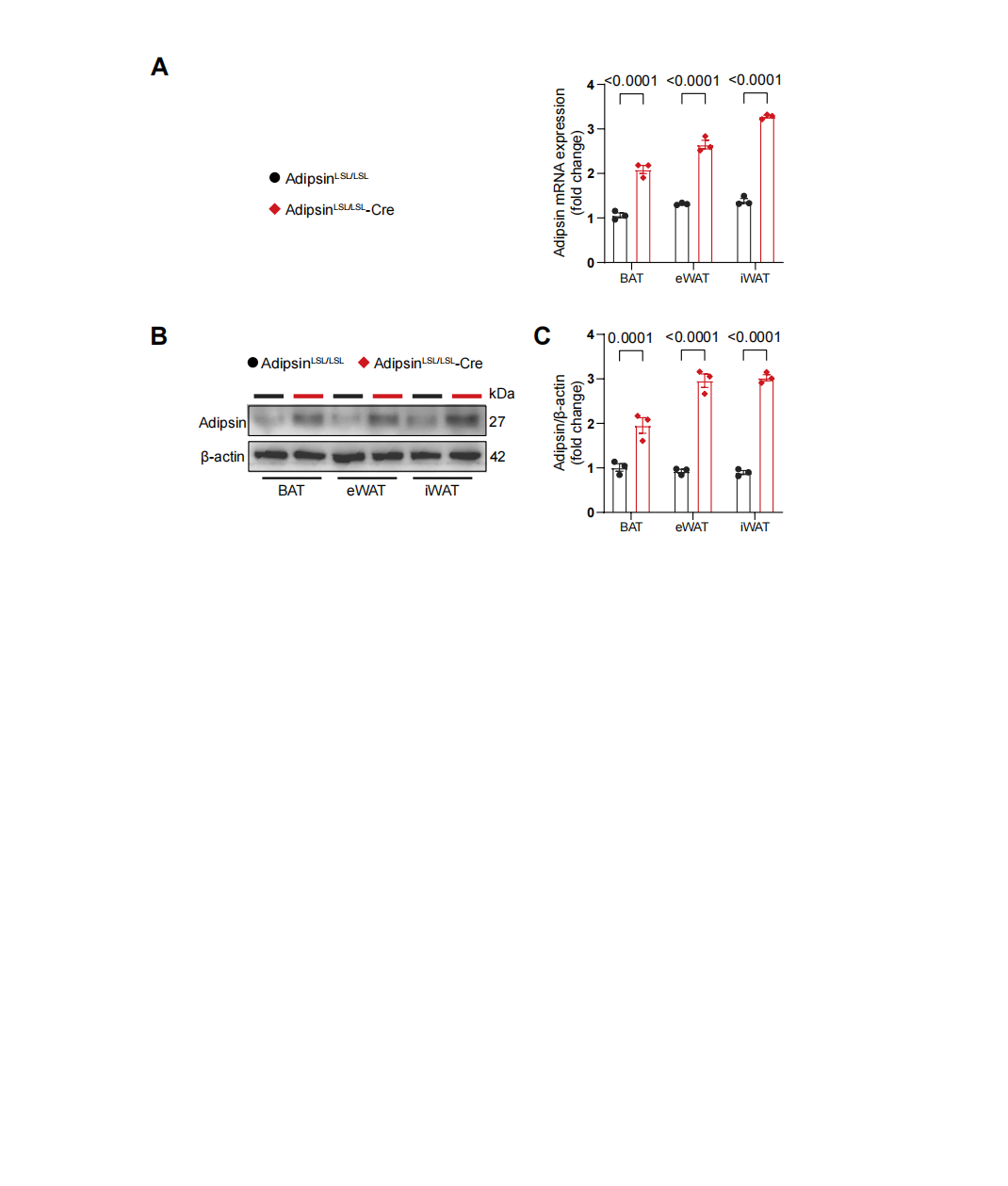


**Figure S2 Changes in Adipsin levels. (A)** Adipsin mRNA levels were quantified in different types of adipose tissues. BAT, brown adipose tissue; eWAT, epididymal white adipose tissue; iWAT, inguinal white adipose tissue. **(B)** Representative Western blot images of Adipsin levels in different adipose tissues. **(C)** Quantitative analysis of Adipsin levels in Figure S2B. Data were presented as mean ± SEM. Students't‐test was used for statistical analysis.

**Figure S3**


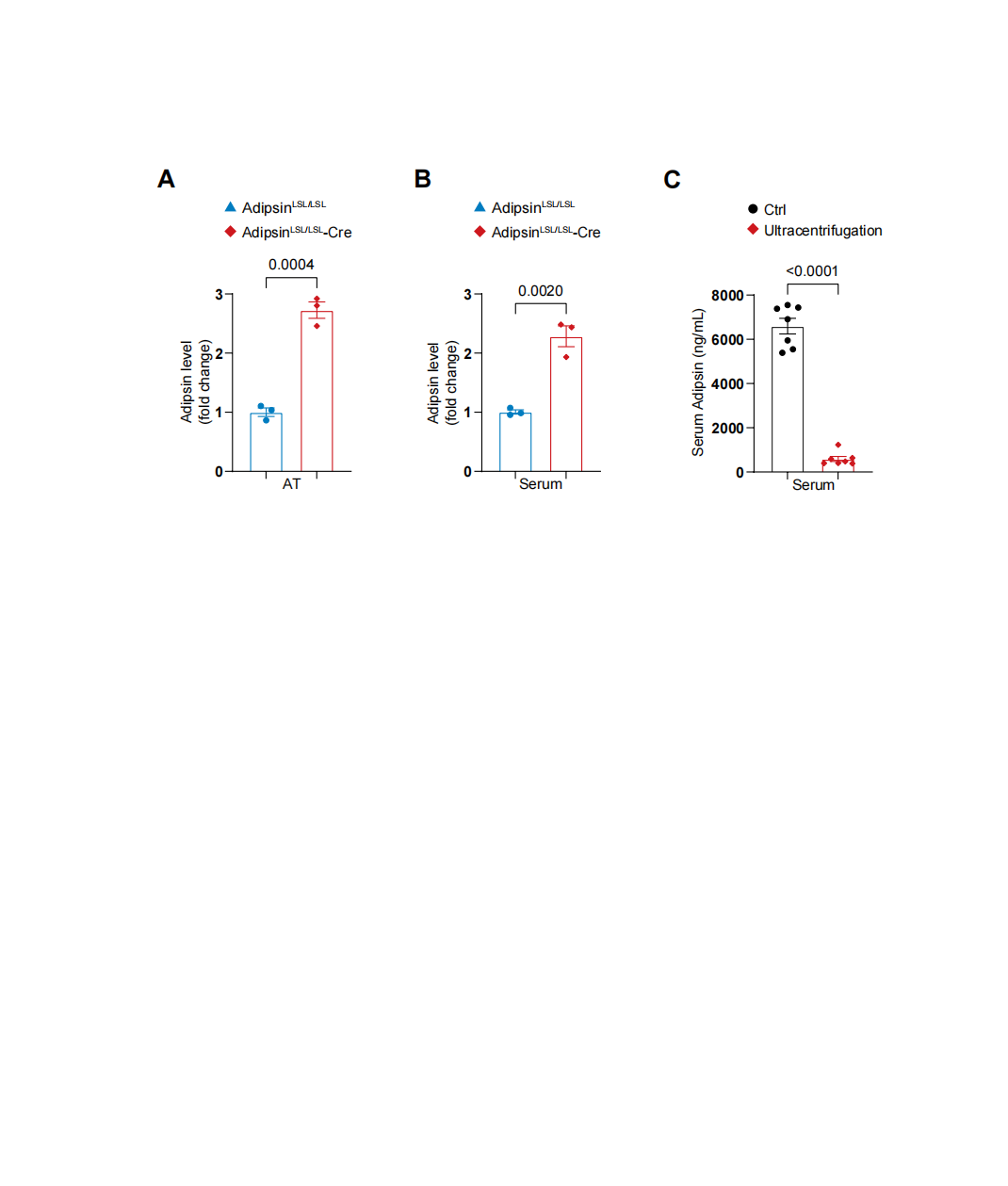


**Figure S3 Adipsin levels in exosomes. (A-B)** Quantitative analysis of Adipsin levels in Figure 4F. **(C)** Serum Adipsin levels determined using ELISA after ultracentrifugation. Data were presented as mean ± SEM. Students't‐test was used for statistical analysis.

**Figure S4**


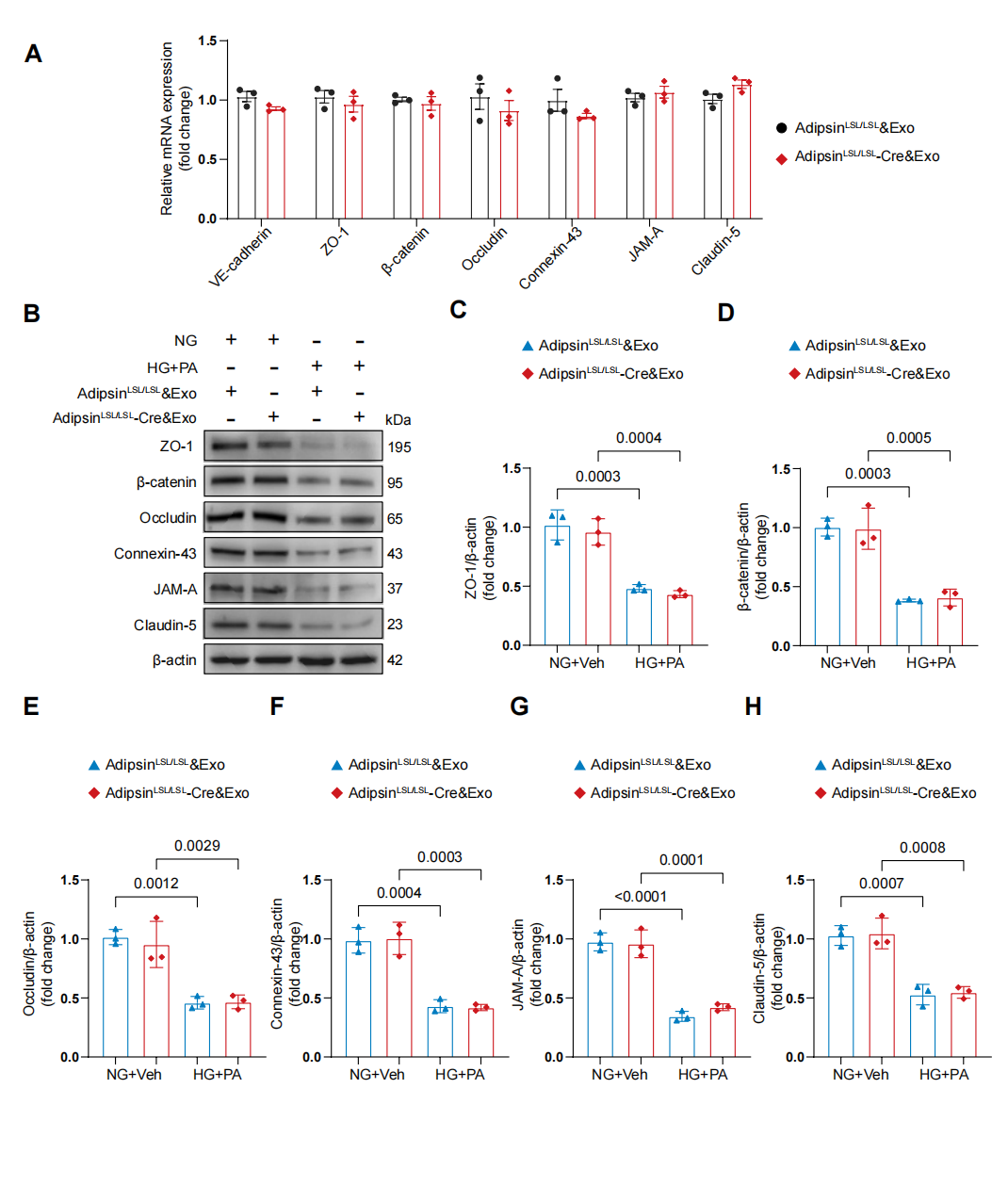


**Figure S4 Expression levels of cell junction molecules are unaffected in CMECs treated with Adipsin^LSL/LSL^&Exosomes or Adipsin^LSL/LSL^-Cre&Exosomes under HG + PA challenge. (A)** Relative mRNA levels of cell junction-associated proteins quantified in different groups. **(B)** Representative Western blot images of cell junction-associated proteins in different groups. **(C-H**) Quantitative analysis of Western blot images from three independent experiments in Figure S4B. Data were presented as mean + SEM. For A, students't‐test was used for statistical analysis. For C-H, one-way ANOVA was used for statistical analysis.

**Figure S5**


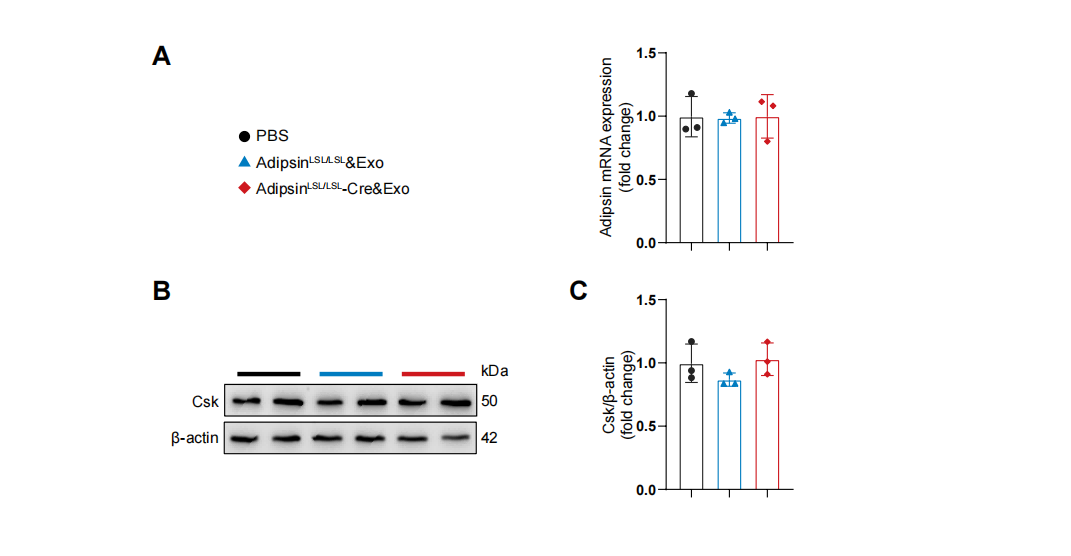


**Figure S5 Adipsin^LSL/LSL^-Cre&Exosomes administration has no effects on Csk expression in CMECs.** **(A)** Csk mRNA levels were quantified in different groups. **(B)** Representative Western blot images of Csk levels in different groups. **(C)** Quantitative analysis of Csk levels in Figure S5B. Data were presented as mean ± SEM. One-way ANOVA was used for statistical analysis.
