# Supplementary material for: Adipsin alleviates cardiac microvascular injury in diabetic cardiomyopathy through Csk-dependent signaling mechanism
Source: BMC Med. 2023 May 26;21:197. doi: 10.1186/s12916-023-02887-7 (PMC10224320; doi:10.1186/s12916-023-02887-7)

**Additional file 3**

**Original Western blots**

**Fig. 1D**

Adipsin


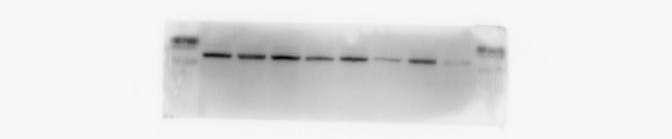


TRF


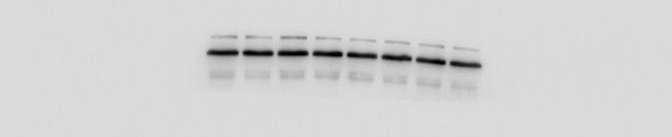


**Fig. 1G**

Adipsin


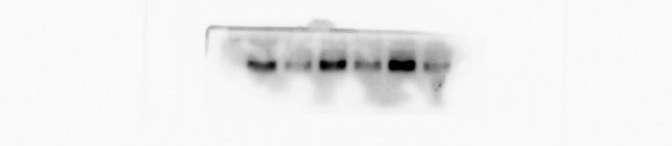


β-actin


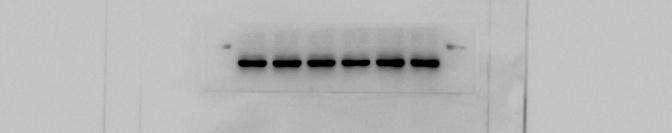


**Fig. 2A**

Adipsin


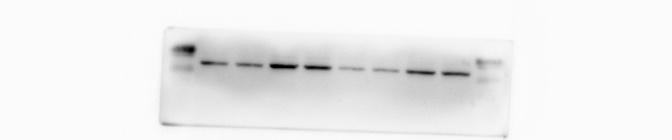


TRF


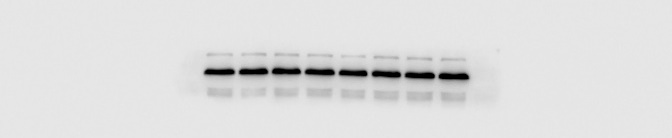


**Fig. 2A**

Adipsin


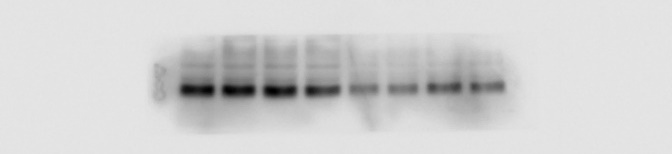


β-actin


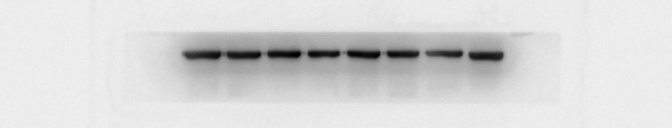


**Fig. 4C**

TSG101

**
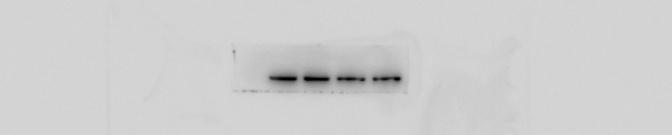
**

CD9

**
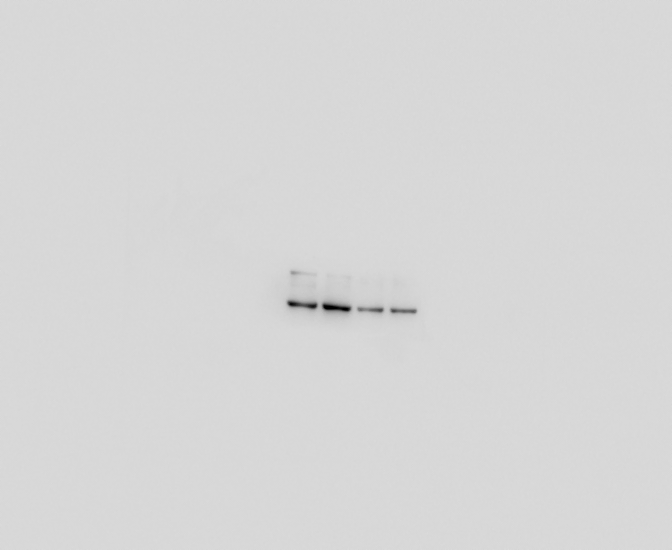
**

CD81

**
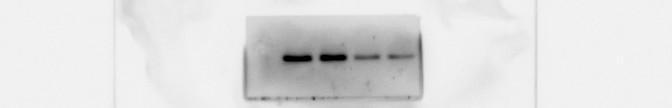
**

Calnexin

**
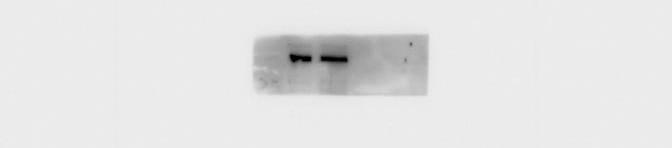
**

**Fig. 4F**

Adipsin

**
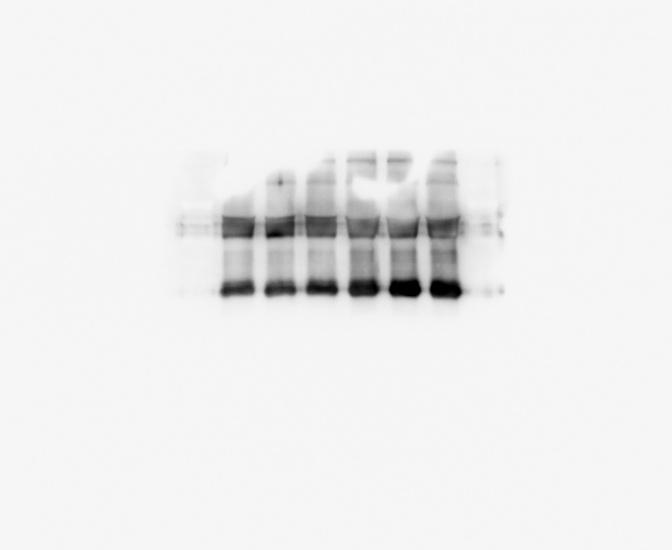
**

Adipsin

**
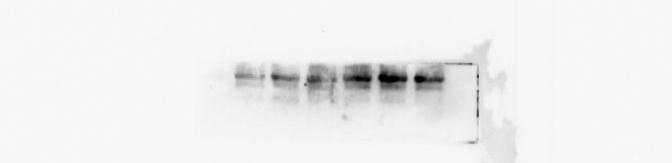
**

**Fig. 7C**

VE-cadherin
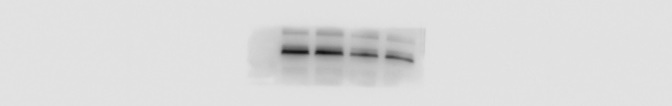


VE-cadherin (Tyr685)


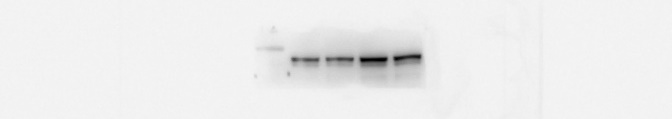


VE-cadherin (Tyr731)


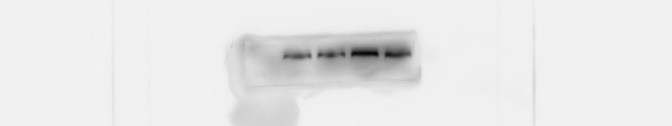


β-actin


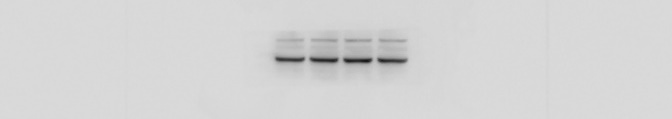


**Fig. 7G**

VE-cadherin (Total)


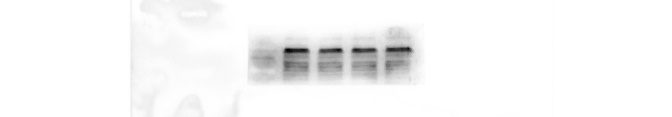


VE-cadherin (Mem)


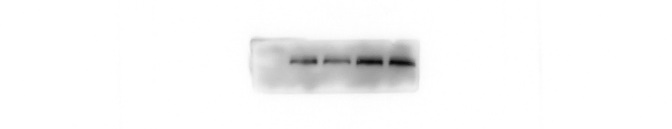


VE-cadherin (Tyr685)


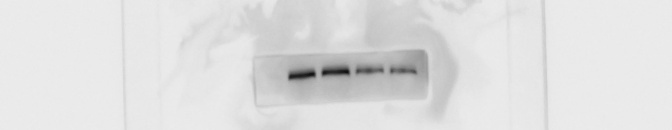


VE-cadherin (Tyr731)


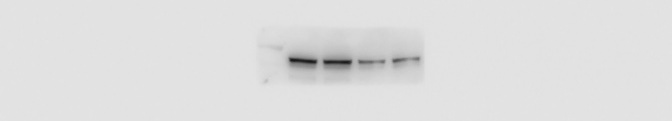


Src (Total)


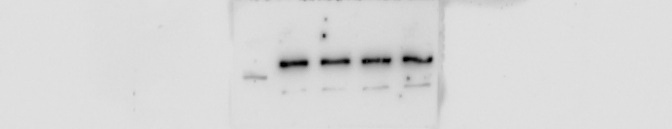


Src (Tyr416)


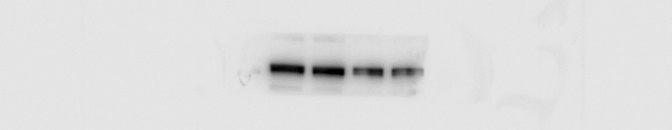


β-actin


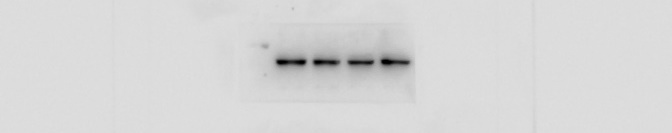


**Fig. 8C**

Csk


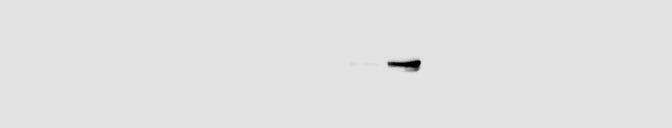


Adipsin


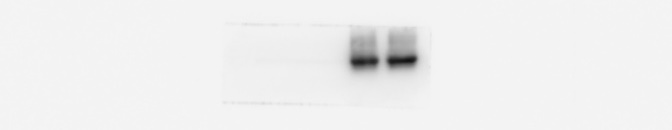


Csk


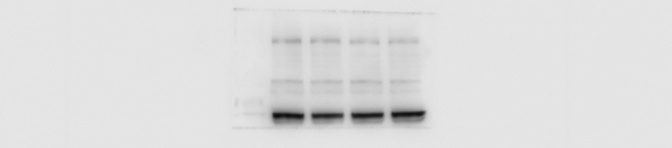


Adipsin


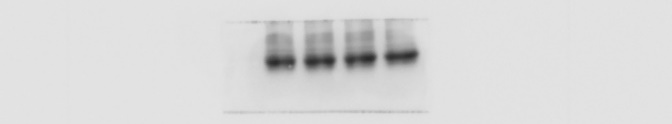


**Fig. 8G**

VE-cadherin (Total)


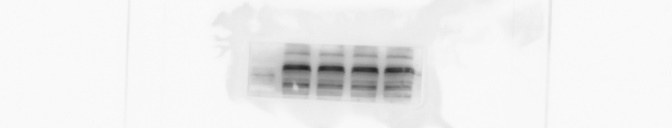


VE-cadherin (Mem)


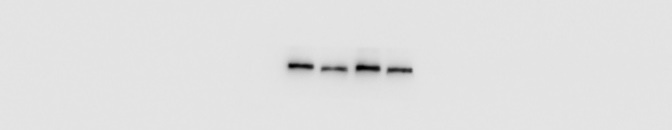


VE-cadherin (Tyr685)


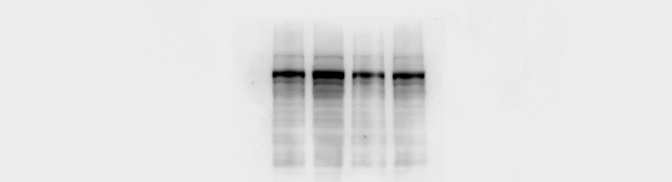


VE-cadherin (Tyr731)


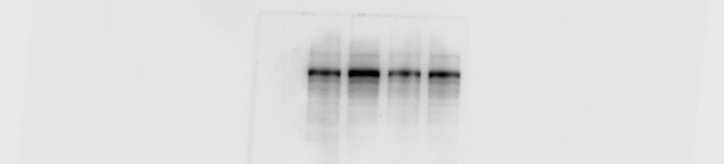


Src


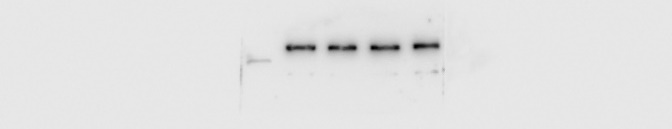


Src (Tyr416)


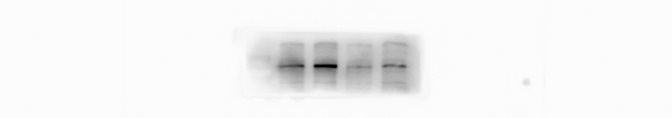


β-actin


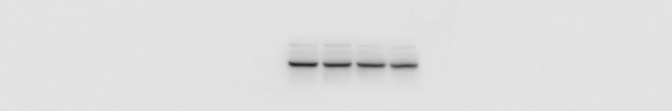


**Additional File 2：Fig. S2B**

Adipsin


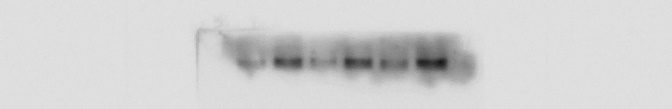


β-actin


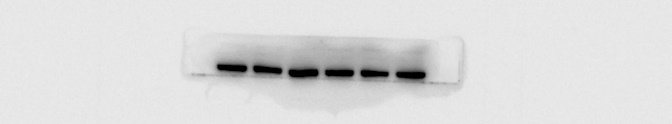


**Fig. S4B**

ZO-1


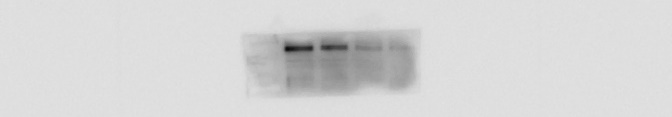


β-catenin


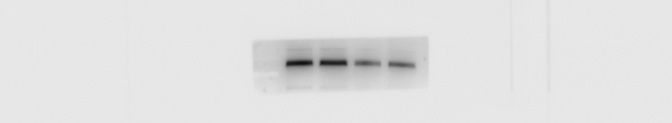


Occludin


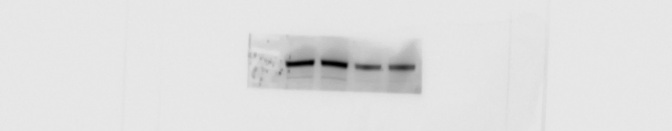


Connexin-43


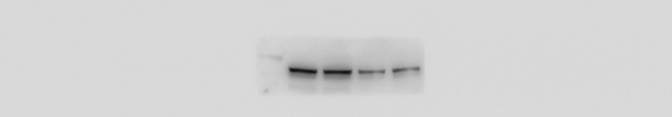


JAM-A


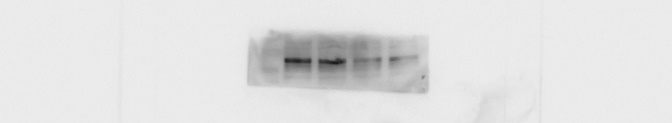


Claudin-5


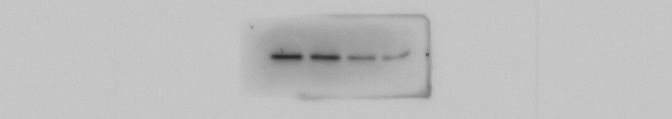


β-actin


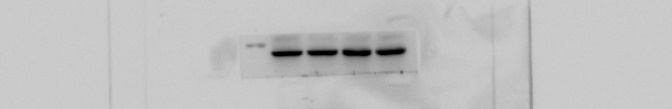


**Fig. S5B**

Csk


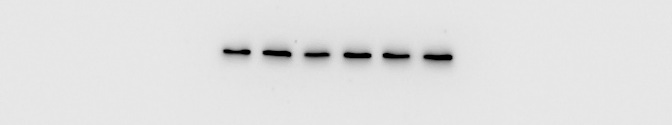


β-actin


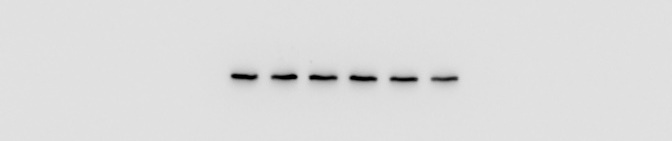

Supplement: Supplementary file 3 — Additional file 3. Original data of Western blots imaging. [file 12916_2023_2887_MOESM3_ESM.docx]
